# Jdp2 is a spatiotemporal transcriptional activator of the AhR via the Nrf2 gene battery

**DOI:** 10.1186/s41232-023-00290-6

**Published:** 2023-08-18

**Authors:** Kenly Wuputra, Ming-Ho Tsai, Kohsuke Kato, Chia-Chen Ku, Jia-Bin Pan, Ya-Han Yang, Shigeo Saito, Chun-Chieh Wu, Ying-Chu Lin, Kuang-Hung Cheng, Kung-Kai Kuo, Michiya Noguchi, Yukio Nakamura, Tohru Yoshioka, Deng-Chyang Wu, Chang-Shen Lin, Kazunari K. Yokoyama

**Affiliations:** 1 Graduate Institute of Medicine, Kaohsiung Medical University, Kaohsiung, 80708 Taiwan; 2 Regenerative Medicine and Cell Therapy Research Center, Kaohsiung Medical University, Kaohsiung, 80708 Taiwan; 3 Cell Therapy and Research Center, Kaohsiung Medical University Hospital, Kaohsiung, 80756 Taiwan; 4 Department of Infection Biology, Graduate School of Comprehensive Human Sciences, the University of Tsukuba, Tsukuba, 305-8577 Japan; 5 Division of General & Digestive Surgery, Department of Surgery, Kaohsiung Medical University Hospital, Kaohsiung, 80756 Taiwan; 6 Saito Laboratory of Cell Technology, Yaita, Tochigi 329-1571 Japan; 7 Department of Pathology, Kaohsiung Medical University Hospital, Kaohsiung, 80756 Taiwan; 8 School of Dentistry, Kaohsiung Medical University, Kaohsiung, 80708 Taiwan; 9 Department of Biological Sciences, National Sun Yat-Sen University, Kaohsiung, 80424 Taiwan; 10 Cell Engineering Division, BioResource Research Center, Tsukuba, Ibaraki 305-0074 Japan; 11 Division of Gastroenterology, Department of Internal Medicine, Kaohsiung Medical University Hospital, Kaohsiung, 80756 Taiwan

**Keywords:** Antioxidation, Aryl hydrocarbon receptor, Dioxin responsive element, Nuclear factor (erythroid-derived 2)-like 2 transcription factor, Jun dimerization protein 2, Oxidative stress, Reactive oxygen species, Transcriptional regulation

## Abstract

**Background:**

Crosstalk between the aryl hydrocarbon receptor (AhR) and nuclear factor (erythroid-derived 2)-like 2 (Nrf2) signaling is called the “AhR–Nrf2 gene battery”, which works synergistically in detoxification to support cell survival. Nrf2-dependent phase II gene promoters are controlled by coordinated recruitment of the AhR to adjacent dioxin responsive element (DRE) and Nrf2 recruitment to the antioxidative response element (ARE). The molecular interaction between AhR and Nrf2 members, and the regulation of each target, including phase I and II gene complexes, and their mediators are poorly understood.

**Methods:**

Knockdown and forced expression of AhR–Nrf2 battery members were used to examine the molecular interactions between the AhR–Nrf2 axis and *AhR* promoter activation. Sequential immunoprecipitation, chromatin immunoprecipitation, and histology were used to identify each protein complex recruited to their respective *cis*-elements in the *AhR* promoter. Actin fiber distribution, cell spreading, and invasion were examined to identify functional differences in the AhR–Jdp2 axis between wild-type and *Jdp2* knockout cells. The possible tumorigenic role of Jdp2 in the AhR–Nrf2 axis was examined in mutant *Kras-Trp53*-driven pancreatic tumors.

**Results:**

Crosstalk between AhR and Nrf2 was evident at the transcriptional level. The *AhR* promoter was activated by phase I ligands such as 2,3,7,8-tetrachlorodibenzo-*p*-dioxin (TCDD) through the AhR–Jdp2–Nrf2 axis in a time- and spatial transcription-dependent manner. Jdp2 was a bifunctional activator of DRE- and ARE-mediated transcription in response to TCDD. After TCDD exposure, Jdp2 activated the *AhR* promoter at the DRE and then moved to the ARE where it activated the promoter to increase reactive oxygen species (ROS)-mediated functions such as cell spreading and invasion in normal cells, and cancer regression in mutant *Kras-Trp53*-driven pancreatic tumor cells.

**Conclusions:**

Jdp2 plays a critical role in *AhR* promoter activation through the AhR–Jdp2–Nrf2 axis in a spatiotemporal manner. The AhR functions to maintain ROS balance and cell spreading, invasion, and cancer regression in a mouse model of mutant *Kras–Trp53* pancreatic cancer. These findings provide new insights into the roles of Jdp2 in the homeostatic regulation of oxidative stress and in the antioxidation response in detoxification, inflammation, and cancer progression.

**Supplementary Information:**

The online version contains supplementary material available at 10.1186/s41232-023-00290-6.

## Background

The US National Toxicology Program has reported that chronic exposure of rats for 2 years to persistent xenobiotics, such as 2,3,7,8-tetrachlorodibenzo-*p*-dioxin (TCDD), induces arteriolar inflammation and remodeling in mesenteric and pancreatic vascular beds [1, 2]. The aryl hydrocarbon receptor (AhR) is activated by these xenobiotics, environmental disruptors, metabolites, bacteria, and inflammatory substances, which can cause multiple effects including inflammation and apoptosis during development [2, 3]. After exposure to the phase I enzyme–ligand complex, the AhR is shuttled into the nucleus by dimerizing with the AhR nuclear translocator (Arnt = hypoxia-inducible factor1β), which subsequently binds to the dioxin responsive element (DRE; 5′-T/GnGCGTG-3′) with a minimum core sequence of 5′-GCGTG-3′ in the promoter region of genes that encode phase I enzymes, such as cytochrome P4501a1 (Cyp1a1), Cyp1a2, and Cyp1b1 [4–7]. Genome-wide analyses of AhR- and Arnt-binding sites have shown overlapping profiles, and these analyses have provided evidence of the importance of the heterodimerization complex in DNA binding [8] and that ligand binding does not alter this specificity [9].

The expression of genes that encode phase II enzymes, such as glutathione S-transferases (GSTs) and UDP-glucuronosyltransferases (UGTs), is known to be regulated in an AhR-dependent manner and to recruit the antioxidative response element (ARE; 5′-G/ATGACNNNGC-3′). The ARE is recognized by nuclear factor (erythroid-derived 2)-like 2 (Nrf2), which is the master regulator of the cellular antioxidant response [8]. Upon exposure to oxidative stress, dissociation of Nrf2 from the Kelch-like ECH-associated protein 1 (Keap1) induces the shuttling of Nrf2 into the nucleus, where it binds to members of the small Maf protein family (MafK, MafF, and MafG) to form a transcriptionally active complex that induces ARE-dependent responses [10–12].

Exposure to AhR ligands, such as dioxins, polycyclic aromatic hydrocarbons, and flavonoids, results in the activation of the genes that encode phase I enzymes and is followed by the Cyp-mediated production of reactive oxygen species (ROS) and electrophilic metabolites [13]. This process also induces the antioxidative response by activating phase II enzymes, which maintain ROS balance through activation by the AhR–Arnt complex of the antioxidative response regulator Nrf2 [11]. Both the AhR and Nrf2 coordinately regulate the genes encoding phase II enzymes such as NAD(P)H quinone oxidoreductase 1 (NQO1), UGT1A10, and heme oxygenase 1 [14–16]. The crosstalk between AhR and Nrf2 signaling in human keratinocytes and liver cells is called the “AhR–Nrf2 gene battery” [17, 18]. The Nrf2-dependent phase II gene promoters are controlled by recruitment of the AhR to the adjacent DRE and recruitment of Nrf2 to the ARE in a coordinated fashion [17, 18]. However, the molecular interaction between AhR and Nrf2 signaling, and the precise regulation of each target, including phases I and II genes, are still poorly understood.

The interaction between the AhR and Nrf2 signaling pathways plays a critical role in protecting against toxic materials through the AhR-dependent detoxification reaction, which maintains ROS balance. ROS are toxic substances produced by aerobic metabolism that can be harmful to cells by inducing DNA damage, cell senescence, or cell death [17, 19]. ROS balance is maintained by oxidative stress responses and antioxidation reactions; for example, the Nrf2-mediated reaction is representative of the latter [20]. By contrast, antioxidation through Nrf2 signaling is necessary to strike a balance between ROS levels and in the detoxification response [21]. High ROS levels are thought to be detrimental to the control of oxygen toxicity, whereas physiologically low ROS levels induced by antioxidative processes serve as an intracellular signaling mechanism [22, 23]. The control of ROS provides a cellular defense mechanism against exogenous and endogenous toxicants and stresses; this process is regulated by the AhR and Nrf2 [24]. However, the links between these signaling pathways and the mediators that control both phase I and phase II enzyme systems have not been elucidated.

Jun dimerization protein 2 (Jdp2), a member of the activator protein 1 (AP-1) family/activation transcription factor family of transcription factors, participates in transcriptional repression via multiple mechanisms that include DNA binding, completion, or inactivation of the formation of heterodimers with other members of the AP-1 family, recruitment of histone deacetylase-3, inhibition of histone acetylation, and direct regulation of chromatin assembly [25–27]. Jdp2 is also a cofactor of the Nrf2‒MafK complex, which regulates ARE and ROS production. Jdp2 binds directly to the core sequence of the ARE, and its association with Nrf2 and MafK increases their DNA-binding activity to the ARE and the transcription of genes encoding antioxidant and detoxification enzymes in mouse embryonic fibroblasts (MEFs) [28, 29].

Reagents that induce oxidative stress stimulate the intracellular accumulation of ROS. For example, a reduced environment in mitochondria can protect against damage caused by ROS production by decreasing the probability of single electron escape to form O_2_^–^ radicals. Jdp2 suppresses ROS generation by modulating the reduced environment. By contrast, control of the gene that encodes the AhR, which is mediated by Jdp2, has not been clarified in terms of its relationship with the phase II enzyme factor Nrf2 and the mediator transcription factor Jdp2. Jdp2 seems to be involved in phase I and phase II enzyme reactions.

Here, we report that the crosstalk between the genes encoding phase I and phase II enzymes is evident at least at the transcriptional level. *AhR* promoter activity induced by phase I ligands, such as TCDD, is regulated by antioxidation transcription factors, such as Jdp2 and Nrf2, in a time- and spatial-dependent manner [29]. Our findings suggest that Jdp2 may be a bifunctional activator of DRE and ARE elements in response to TCDD in MEFs. We found that Jdp2 was present in the region proximal to the promoter/enhancer regions of the AhR during the initial stage of the response to TCDD and interacted with the AhR, Arnt, and MafK to increase *AhR* promoter activity. Here, we also show that, in the initial phase of TCDD exposure, Jdp2 is an upstream regulator of *AhR* promoter elements together with Nrf2 on DRE2 and that ARE1 elements interact with the AhR, Arnt, and Nrf2 in the *AhR* promoter region. Taken together, our findings suggest that the transcription factor Jdp2 plays a key role in AhR and Nrf2 activation to maintain ROS balance, cell migration and spreading, and cancer regression in a mouse model of *Kras–Trp53* pancreatic cancer [30]. Jdp2 may act as a decisive factor during ROS regulation that involves AhR–Arnt phase I and Nrf2–small Maf phase II complexes.

## Methods

### Animals, cell culture, and reagents

The animal welfare guidelines for the care and use of laboratory animals were those published by the Animal Care Committee of the RIKEN BioResource Research Center (BRC) in Japan (Kiteisv.intra.riken.jp/JoureiV5HTMLContents/act/print/print110000514.htm), the National Laboratory Animal Center in Taiwan (106,022), and Kaohsiung Medical University in Taiwan (106,189; 107,128; 108,244). All animal experiments were performed in accordance with these approved guidelines.

The strategy for “*Jdp2*^*–/–*^ knockout” was as described previously [31, 32]. Primary MEFs were established from the embryos of WT and *Jdp2*^*−/−*^ littermates. The cells were cultured in DMEM (High Glucose; HyClone Laboratory Inc., Seattle, WA, USA) containing 10% fetal bovine serum (FBS; Invitrogen, Grand Island, NY, USA), as described previously [31, 32]. Human HepG2, 293T, and MCF7 cells were obtained from the RIKEN BRC Cell Bank (Tsukuba, Japan). The mouse 2546 pancreatic cancer cell line was generated and cultivated as described previously [30]. DMSO, BaP, tBHQ, sulforaphane, FICZ, and l-kynurenine were obtained from Sigma-Aldrich (St. Louis, MO, USA). TCDD was purchased from AccuStandard (New Haven, CT, USA).

### Vector and virus construction and antibodies

*pcDNA-Nrf2*, *pcDNA-MafK*, *pcDNA-Jdp2*, and *pcDNA-Jdp2A139C* were obtained from RIKEN BRC, amplified by PCR, and cloned into a *pCMV_S-FLAG* vector using the respective restriction sites [29]. *pQCXIN-CA-AhR-EGFP* and *pCYP1b*-luciferase were kind gifts from Dr. Yoshiaki Fujii-Kuriyama of Tsukuba University (Tsukuba, Japan). PCR-amplified DNA fragments were inserted into the *pCMV_S-FLAG* vector. The coding region of *Jdp2* was inserted at the *Xho*I*/Eco*RI site of the *pPyCAG-BstXI-IRES-Zeocin-pA* vector (kindly provided by Dr. H. Niwa, Kumamoto University, Kumamoto, Japan) to generate *pPyZmJdp2*. Lentiviral vectors *pCAG-HIVgp*, *pCMV-VSV-G-RSV-Rev*, and *CSII-CMV-MCS-IRES2-Bsd* were obtained from the RIKEN BRC DNA Bank (http://www.brc.riken.jp/lab/cfm/Subteam_for_Manipulation_of_Cell_Fate/Lentiviral_Vectors.html). All antibodies used in this study are listed in Table S1.

### Immunocytochemistry

Cells were fixed in 4% formaldehyde for 10 min, washed with phosphate-buffered saline (PBS), incubated with blocking solution containing 10% FBS and 0.1% Triton X-100 in PBS for 15 min, and incubated overnight with the primary antibodies; Rabbit anti-Jdp2 (a gift from Aronheim); Goat anti-AhR (Santa Cruz Biotechnology, Dallas, TX, USA; SC-8088); Rat anti-Nrf2 (Cell Signaling Technology, Danvers, MA, USA; CST # 14,596), After washing with PBS-Tween 20 (PBS-0.05% Tween), the cells were incubated for 1.5 h with the following secondary antibodies: Alexa Fluor® 594-labeled goat anti-rabbit IgG (Thermo Fisher Scientific, Waltham, MA, USA; A-11037), Alexa-Fluor® 488 conjugated Rabbit anti-Goat IgG (Thermo Fisher Scientific; A-11078), and Alexa Fluor® 647-labeled goat anti-rat IgG (H + L) (Cell Signal Technology; #4418). The cells were then processed using 4,’6-diamino-2-phenylindole (DAPI) to visualize cell nuclei (1:3000; 5 mg/mL stock in DMSO; Sigma-Aldrich). Cells were mounted on slides using ProLong® Gold antifade mountant (Molecular Probes, Thermo Fisher Scientific; P36934), and cell immunofluorescence was visualized using an Olympus FV1000 confocal laser scanning microscope (Olympus, Tokyo, Japan).

### SDS-PAGE, immunoprecipitation, and Western blotting

Sodium dodecyl sulfate (SDS) polyacrylamide gel electrophoresis (PAGE), IP, and Western blotting assays were performed as described previously [33]. Briefly, cells were lysed on ice with RIPA buffer (50 mM Tris–HCl, pH 7.4, with 1% Nonidet P-40, 0.25% sodium deoxycholate, and 1 mM ethylenediaminetetraacetic acid (EDTA)) containing protease and phosphatase inhibitors (1 mM phenylmethyl sulfonyl fluoride, 1 mM Na_3_VO_4_, 1 mM NaF, and 1 μg/mL of aprotinin, leupeptin, and pepstatin A each; all added immediately before use). In some experiments, cell lysates were centrifuged at 500 × *g* for 5 min at 4 °C to prepare nuclei and cytosol fractions using NE-PER Nuclear and Cytoplasmic Extraction Reagent (Thermo Fisher Scientific). All lysates were fractionated by SDS-PAGE and transferred to a 0.45 μm Immobilon®-P polyvinylidene difluoride (PVDF) membrane (Merck, Darmstadt, Germany; IPVH00010) for 1 h at 100 V (fixed) at 10 °C using a TE22 Mighty Small Transfer Tank system (BioCompare, San Francisco, CA, USA). Blots were stained with Ponceau S (Merck; P17170) to monitor the transferred protein amounts. PVDF membranes were then probed with the primary and secondary antibodies (Table S2). The results were analyzed using a ChemiDoc XRSPlus instrument (Bio-Rad, Hercules, CA, USA). IP was performed using antibody-coated protein A/G beads as described previously [34].

### ChIP assay

For the ChIP assay, cells were collected with a plastic scraper and fixed in 1% formaldehyde in PBS for 8 min at room temperature with rotation, 0.125 M glycine was added, and the solution was incubated at room temperature for 5 min to quench the crosslinks. The cells were harvested in cold PBS with protease inhibitors and washed three times for 5 min each at 4 °C. The collected cells were lysed by pipetting the pellet with 750 μL of SDS lysis buffer (50 mM Tris–HCl pH 8.0, 10 mM EDTA, 1% SDS) with proteinase inhibitors and incubated on ice for 30 min. The cells were then sonicated using a SONICS VC50 instrument (Sonics & Materials Inc., New Town, CT, USA) for 25 min (5 s on, 15 s off) on ice, which sheared the DNA to an average size of 350 bp. The antibodies of interest or IgG negative control (4 μg) were added, and the cells were incubated overnight. Precleared protein A/G agarose beads 1:1 (Merck Millipore, Burlington, MA, USA) were added to the samples, and the samples were incubated at 4 °C for 3 h. The beads were washed using the four following buffers: low-salt buffer (0.1% SDS, 0.1% Triton X-100, 150 mM NaCl, 2 mM EDTA, 20 mM Tris–HCl pH 8.0); high-salt buffer (0.1% SDS, 0.1% Triton X-100, 500 mM NaCl, 2 mM EDTA, 20 mM Tris–HCl pH 8.0); IP wash buffer (0.5 M LiCl, 1% NP-40, 1% deoxycholic acid, 100 mM Tris–HCl pH 9.0); and Tris–EDTA buffer (10 mM Tris–HCl pH 8.0, 1 mM EDTA). Samples were eluted from the beads and reverse crosslinked using 0.3 M of NaCl at 65 °C overnight, after which proteinase K was used to release DNA, and phenol/chloroform/isoamyl (25:24:1) was used to isolate the DNA fragments. Data were analyzed using a real-time PCR assay (10 mM Tris–HCl pH 8.0, 1 mM EDTA). The antibodies used were to the AhR (3 μg, sc-8088; Santa Cruz Biotechnology, Inc., Dallas, USA), Nrf2 (3 μg, sc-722; Santa Cruz Biotechnology, Inc., Dallas, USA), and JDP2 (3 μg; from Dr. A. Aronheim, Technion-Israel Institute of Technology, Haifa, Israel). The primers for each fragment of detection are shown in Table S3.

### Construction of AhR promoter plasmids and site-directed mutagenesis

*AhR* promoter regions were cloned from *Mus musculus* strain C57BL/6J chromosome 12, GRCm38.p4 C57BL/6J 35535598 to 35,536,615, using a KAPA HiFi PCR kit (Kapa Biosystem Inc., Bath, UK) with the following primers: 5′-ATAGGTACCGGATCCCCTCTTCTCCTTCT-3′ and 5′-ATACTCGAGGCTGCTCATGGTG-3′ with *Kpn*I or *Xho*I sites added on the 5′-end. The total length of 1947 bp was cloned into the *pGL4.1* plasmid (Promega, Madison, WI, USA). The location and orientation of the construct were confirmed using endonuclease restriction analysis and next-generation sequencing. The binding sites of the *AhR* promoter region were predicted using ALGGEN-PROMO (http://alggen.lsi.upc.edu). A Quick-Change Lightning Site-Directed Mutagenesis Kit (Agilent Technologies, Santa Clara, CA, USA) was used to change individual sites of the *AhR* promoter regions (Table S3).

### Transient transfection and luciferase reporter assay

Cells were plated into 24-well plates (4 × 10^4^ cells/well) and cultured for 24 h. The cells were then cotransfected with 500 ng of the *AhR* or Cytochrome P450 Family 1 Subfamily B Member 1 (*CYP1B1*) (− 2299/ + 25) luciferase plasmid and 10 ng of the *pRL-CMV* plasmid encoding *Renilla* luciferase using Lipofectamine 2000 (Invitrogen) or polyethylenimine (linear, molecular weight 25,000; Polysciences, Warrington, PA, USA; Cat# 23,966). *pGL3-basic* plasmid containing the 5′-flanking-region from − 2299 to + 25 of *CYP1B1* was kindly provided by Drs. M. Nakajima (Kanazawa University, Kanazawa, Japan) and K. Fujii-Kuriyama (Tsukuba University, Tsukuba, Ibaraki, Japan). The total amount of transfected DNA was kept constant at 1 μg/well by the addition of a *pBluescript II SK +* control plasmid (Addgene, Watertown, MA, USA). The transfected cells were treated with DMSO or TCDD for the indicated times and harvested 48 h after the transfection. Luciferase activity was measured using a dual-luciferase reporter assay system according to the manufacturer’s instruction using a GloMax20/20 Luminometer (Promega). The reporter activity was calculated as relative luciferase activity (firefly luciferase/*Renilla* luciferase) and is expressed as fold induction compared with the empty vector in WT MEFs. All measurements were performed in duplicate, and the values are expressed as mean ± standard error of the mean (SEM) from at least three independent experiments.

### shRNA- and siRNA-mediated gene knockdown

shRNA lentiviruses against mouse *AhR*, *Nfe2l2 (Nrf2)*, *MafK*, *Arnt*, *GFP*, and *Jdp2* were obtained from the siRNA Core Center at Academia Sinica (Taipei, Taiwan). The predesigned ON-TARGETplus SMARTpool siRNA against mouse *AhR*, *Arnt*, *Ahrr*, *MafK*, and *Nfe2l2*, and a control scrambled siRNA were purchased from GE Dharmacon (Austin, TX, USA). MEFs were seeded into a six-well plate (for Western blotting) or 24-well plate (for the luciferase reporter assay) and transfected in the presence of 20–40 nM of either siRNA or negative control RNA in a final volume of 0.5 mL (24-well plate) or 2 mL (six-well plate) of OPTI-MEM with Lipofectamine RNAiMAX (both from Invitrogen). shRNA was transfected into MEFs at a multiplicity of infection of 10. After 24 h, fresh culture medium containing 10% FBS was added, the cells were transfected with the luciferase plasmids, and the luciferase reporter assay was performed as described above. To confirm the knockdown efficiency of shRNA and siRNA, cells were harvested at 48 and 72 h after shRNA infection and siRNA transfection, respectively, and analyzed by immunoblotting and other methods.

### Isolation of RNA and qPCR

Total RNA was purified using a PureLink™ RNA Mini Kit (Invitrogen). RNA was reverse transcribed to cDNA using SuperScript III Reverse Transcriptase (Invitrogen). Real-time PCR was performed on a StepOne or ABI7500 PCR instrument (Applied Biosystems, Foster City, CA, USA) using Applied Biosystems Fast SYBR® Green Master mix in 20 μL reaction volumes. The threshold cycle *Ct* values were averaged from technical duplicates. The transcript level of each gene was normalized to that of *Gapdh*. The 2^–ΔΔ*Ct*^ method was used to calculate relative gene expression level, and expression was normalized to the mRNA level in DMSO- or TCDD-treated WT MEFs, which was taken as 1.0. Data are expressed as mean ± SEM of three biological replicates. The forward and reverse primers used were 5′-TAGGAAGAGAAGGAAGCCCATTCA-3′ and 5′-GGTGCCGTTTGGAAGGATTTG-3′ for *Ahrr*; 5′-TGCTGGAGAGGACTGTGTAGA-3′ and 5′-GGTCGAGTCTTGCCTGAGTT-3′ for *Aldh3a*; 5′-TTACGGACATCTTCGGAGCC-3′ and 5′-CCCACAACCTGGTCCAACTC-3′ for *Cyp1b1*; and 5′-ACGAAGGCTGTCTACACCAC-3′ and 5′-CCCGAGAGTTGGCTTCTT CA-3′ for *Tiparp*.

### Coimmunoprecipitation and Western blot analysis

Whole-cell lysates were prepared using PRO-PREP™ protein extraction solution (iNTRON Biotechnology, Gyeonggi-dom, Korea). Subcellular fractionation was performed using an NE-PER® Nuclear and Cytoplasmic Extraction kit (Thermo Fisher Scientific). Protein concentration was measured using the Bradford method with bovine serum albumin as the standard (Bio-Rad). For coimmunoprecipitation, 300 μg of extract was precleared with Protein G Agarose beads (Millipore, Burlington, MA, USA) at 4 °C for 1 h and then incubated with 1 μg of IP antibody or preimmune IgG at 4 °C overnight with rotation. The immune complexes were precipitated with protein G beads and analyzed by Western blotting, as described below. The immunoprecipitated proteins were dissolved in 30 μL of 2 × Laemmli buffer, boiled for 5 min, and analyzed by Western blotting.

To measure p-MLC2, MEFs were deprived of serum overnight, treated with DMSO with or without TCDD for 6 h, and lysed with 10% trichloroacetic acid. Precipitated protein was collected by centrifugation, washed in absolute ethanol three times each for 5 min, and solubilized completely in urea buffer (8 M urea, 20 mM Tris, 23 mM glycine, and 0.2 mM EDTA). Equal amounts of protein were electrophoresed on an acrylamide gel and then transferred to nitrocellulose. After blocking with 5% nonfat dry milk in Tris-buffered saline containing 0.1% Tween 20 for 1 h at room temperature, the membrane was blotted with the primary antibody at 4 °C overnight with shaking and then incubated with the horseradish peroxidase-conjugated secondary antibody for 1 h at room temperature. Immunoreactive bands were visualized with Immobilon Western Chemiluminescent HRP Substrate (Millipore), and images were captured using a ChemiDoc-XRS^+^ apparatus (Bio-Rad) and quantified using Image Lab software (version 4.1). The primary antibodies used were to the following proteins (AhR (1:1000, sc-8088; Santa Cruz Biotechnology, Inc., Dallas, USA), Nrf2 (1:1000, sc-722; Santa Cruz Biotechnology, Inc., Dallas, USA), Arnt (1:1000, CST5537; Cell Signal Technology, Danvers, MA, USA), p-MLC2^Ser19^ (1:1000, CST3671; Cell Signal Technology, Danvers, MA, USA), MLC2 (1:1000, CST3672; Cell Signal Technology, Danvers, MA, USA), JDP2 (1:1000, from Dr. A. Aronheim or sc-367695), MafK (1:1000, sc-477; Santa Cruz Biotechnology, Inc., Dallas, USA), Lamin A/C (1:1000, sc-6215; Santa Cruz Biotechnology, Inc., Dallas, USA), and β-actin (1:5000, sc-81178; Santa Cruz Biotechnology, Inc., Dallas, USA).

### ROS detection by CM-H_2_DCFDA fluorescence and flow cytometry

Cells were cultured in 0.1% gelatin-coated 12-well plates. After treatment with 10 nM TCDD for the indicated times, cells were rinsed with warm Hanks balanced salt solution (HBSS; Gibco Invitrogen, Waltham, MA, USA), and then loaded with 10 μM CM-H_2_DCFDA (C-6827, Life Technologies) in complete growth medium for 30 min at 37 °C in the dark. After loading, the cells were washed twice with HBSS and examined under a Nikon inverted fluorescence microscope. Three to five fields were randomly selected for imaging with a × 10 objective lens and quantified using ImageJ (National Institutes of Health, Bethesda, MD, USA). The value for WT-basal was used to normalize the results and was set at 1.0. For flow cytometry, cells were suspended in HBSS with 10 μM CM-H_2_DCFDA and cultured in the dark at 37 °C for 20 min, and propidium iodide was added after 15 min of incubation. An LSR II flow cytometry (Bio-Rad) with PMT F505 LP bandpass filter was used to detect the CM-H_2_DCFDA signal.

### Measurement of 8-oxo-dGuo, glutathione, NQO1, and MDA levels, and cellular ROS accumulation

The concentration of 8-oxo-dGuo was measured using liquid chromatography–mass spectrometry as described previously [33, 35]. GSH and GSSG concentrations (mmol/mg protein) were calculated from a standard curve using a GSH assay kit (Cayman Chemical Co., Ann Arbor, MI, USA; 703,002) and normalized against the protein concentration. NQO1 activity was measured using a 2,6-dichlorophenolindophenol reduction assay, as described previously [36, 37]. MDA was used as an indicator of lipid peroxidation, and its level was measured using a thiobarbituric acid reactive substance assay kit (Abcam, Cambridge, UK; ab18970) according to the manufacturer’s instructions [34]. The ROS-Glo™ H_2_O_2_ assay (Promega) was used to measure the net intracellular accumulation of ROS. After 2 h of treatment with antioxidants and H_2_O_2_, cells were washed with HBSS twice and incubated with ROS-Glo™ Detection Solution for 20 min, and the fluorescence was detected using a GloMax® fluorometer (Promega) [38].

### Xenograft injection

Cells were cultured in medium as recommended at a density of 1 × 10^6^ cells in a 10-cm dish and transfected with the required overexpression or shutdown vectors using the lentivirus system 48 h before injection. The injection medium was combined with Matrigel Matrix (Corning, Glendale, AZ, USA) as recommended: 1 × 10^5^ cells were injected subcutaneously into severe deficient immunocompetency (SCID) mice; the xenografts were traced 3 weeks after the injection; the xenograft weights were measured in milligrams; and the xenografts were fixed in 4% formaldehyde for biopsy analysis.

### RNA sequencing, gene clustering, and gene categorization

RNA sequencing was performed on an Illumina GAIIX instrument (Illumina Inc., San Diego, CA, USA) using the 50 bp single-end protocol of Welgene Biotech (Taipei, Taiwan) as described previously [39, 40]. RNA sequencing data were deposited in the NCBI BioProject Database (http://www.ncbi.nlm.nih.gov/bioproject) with the accession numbers SUB3541857, SUB3541902, SUB3541913, and SUB3541945.

### Measurement of cell area after immunofluorescence staining of p-MLC2 and actin stress fibers, and a wound-healing assay

Cells were treated with trypsin and replated in eight-well chamber slides precoated with 0.1% gelatin and allowed to spread for 6 h at 37 °C in complete medium containing DMSO (0.1%) or TCDD (50 or 100 nM). Cells were then fixed with 4% formaldehyde for 30 min and permeabilized with 0.2% Triton X-100 in PBS for 15 min at room temperature. After blocking with 5% goat serum in PBS, cells were stained overnight with rabbit anti-p-MLC (1:50, Cell Signaling Technology, Danvers, MA, USA) at 4 °C, washed thoroughly with PBS, and incubated with Alexa Fluor 594 goat anti-rabbit secondary antibody (1:400, Invitrogen) at room temperature for 60 min. To label actin stress fibers, actin was counterstained in green using Alexa Fluor 488-labeled phalloidin (1:80, Invitrogen). Cell nuclei were stained in blue using DAPI. Fluorescence images from five different fields per treatment were acquired using a Nikon epifluorescence microscope. Cell area and fluorescence intensity were quantified using ImageJ software (National Institutes of Health, Bethesda, ML, USA). In other experiments, MEFs were serum starved overnight and then treated with DMSO or TCDD for another 24 h. At the end of treatment, cells were rinsed with PBS, fixed with 4% formaldehyde, and then stained for F-actin and nuclei as described above. For each treatment, at least five randomly selected fields were acquired.

The wound-healing assay was performed using fully confluent MEFs at 1.2 × 10^6^ cells in a six-well dish precoated with 0.1% gelatin. Mitomycin C was added at a concentration of 10 μg/mL, and the cells were incubated for 4 h at 37 °C. Wounds were created using a 1-mL micropipette tip, and the scraped cells were washed from the culture dish with PBS. TCDD (10 or 50 nM) or DMSO as the negative control in culture medium was added to the cells, and the cells were incubated for 24 h. All data were analyzed using ImageJ software.

### Statistical analysis

Data are shown as the mean ± SEM. The results were compared between experimental conditions using GraphPad Prism software (v. 5.0; GraphPad Software, San Diego, CA, USA). For multiple comparisons, one-way analysis of variance (ANOVA) followed by the Tukey post hoc test or two-way ANOVA with the Bonferroni post hoc test was used. Student’s unpaired two-tailed *t* test was used to compare the control and treatment groups. Student’s paired one-tailed *t* test was used to identify each site-directed mutagenesis site in the *AhR* promoter. Mann–Whitney nonparametric median statistical analysis was used to compare cell areas. All differences were significant at *p* ≤ 0.05.

## Results

### Jdp2 deficiency attenuates Cyp1b1 promoter activity in response to TCDD

A potent AhR activator, TCDD is known to be an inducer of the expression of Cyp1b1 and Cyp1a1 in mouse and human cells [41]. We used Western blotting and real-time polymerase chain reaction (qPCR) analysis to examine the activation of AhR target genes, such as *Cyp1a1* and *Cyp1b1*, in wild-type (WT) MEFs and *Jdp2*-deleted MEFs (*Jdp2*^*–/–*^ MEFs) (Supplementary Figure S1A and B). *Cyp1a1* expression did not differ significantly between WT and *Jdp2*^*–/–*^ MEFs, but *Cyp1b1* expression was markedly lower in *Jdp2*^*–/–*^ MEFs than in WT MEFs.

Next, to investigate whether the transcriptional activation of an endogenous AhR target gene, *Cyp1b1*, is affected by Jdp2, promoter luciferase reporter assays were conducted using WT and *Jdp2*^*–/–*^ MEFs. As expected, the *Cyp1b1* reporter was activated by about 16-fold after a 6-h incubation with TCDD in WT MEFs (Supplementary Figure S1C), and this level was maintained until 24 h. A similar trend was observed in *Jdp2*^*–/–*^ MEFs, but the activity was about only one-sixth of that observed at 6 h in WT MEFs (Supplementary Figure S1D). The maximal *Cyp1b1* promoter activity induced by TCDD was attained at a concentration of 10–200 nM in WT MEFs. In *Jdp2*^*–/–*^ MEFs, TCDD also increased *Cyp1b1* promoter activity but only to 50–60% of that observed in its WT counterpart (Supplementary Figure S1E).

Interestingly, *Nfe2l2* silencing, but not the scrambled control, also suppressed TCDD-evoked activation of *Cyp1b1* luciferase. In addition, knockdown of MafK increased the promoter activity of *Cyp1b1* luciferase. These findings suggest that, in WT MEFs, MafK has a negative effect on the promoter activity of *Cyp1b1* (Supplementary Figure S1F). TCDD-evoked *Cyp1b1* promoter activity increased significantly after the addition of 50 ng of Jdp2 to *Jdp2*^*–/–*^ MEFs (Supplementary Figure S1G).

The results of knockdown by small interfering RNA (siRNA) and those obtained in *Jdp2*^*–/–*^ MEFs suggest that Nrf2 and Jdp2 function as an ARE-binding complex, which is required for *AhR* promoter activation, but MafK depletion increases *AhR* promoter activation. These results agree with those of an earlier report that Nrf2 regulates *AhR* transcription and subsequently modulates several downstream events in the AhR signaling cascade, such as the transcriptional control of the xenobiotic metabolism gene *Cyp1b1* [42]. However, our findings here suggest that members of the small Maf family, such as MafK, may be negative repressors of *Cyp1b1*, which contradicts published results [8, 10–12] but is consistent with our previous findings [29, 43].

### Jdp2 is required for the expression of AhR protein

**Fig. 1:**
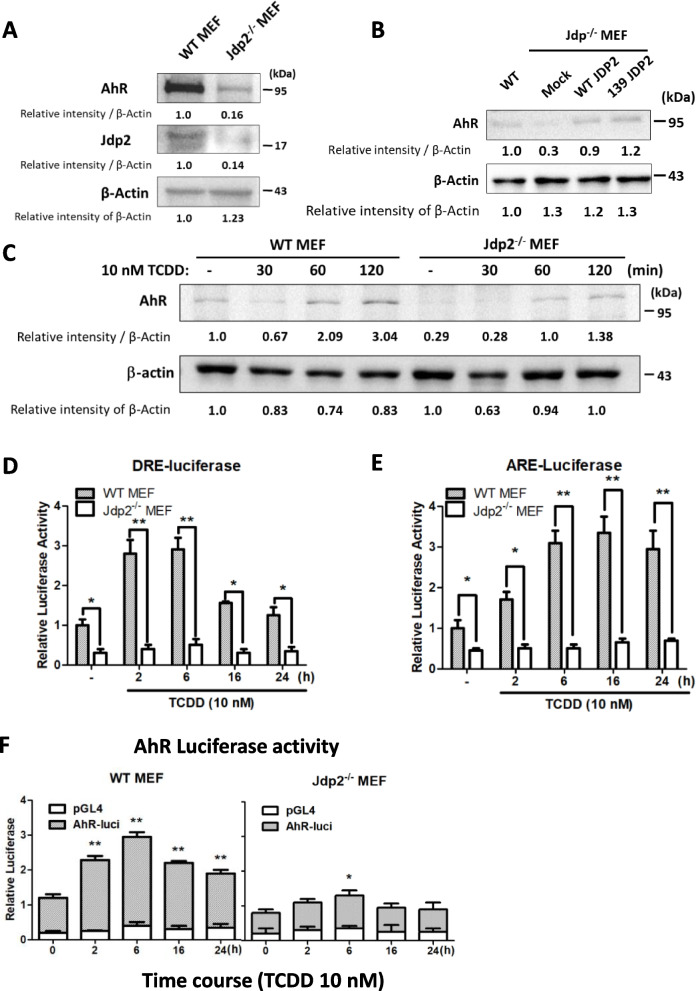
Reduced AhR expression and its promoter activity after deletion of Jdp2. **A** Comparison of the levels of AhR protein in WT and *Jdp2*^*–/–*^ MEFs. **B** WT *Jdp2* rescued AhR expression in *Jdp2*^*–/–*^ MEFs. WT Jdp2: wild-type Jdp2; 139Jdp2 (C139AJdp2): alanine-mutated Jdp2 at position 139 [44]. After selection with G418 and harvesting for testing, AhR expression was measured. **C** Comparison of AhR protein levels in WT and *Jdp2*^*–/–*^ MEFs treated with 10 nM TCDD for indicated time-periods. See Supplementary Figure S8 for the original full-length blot images. The intensity of each band was then quantified, and the relative value was normalized to β-actin. **D** Relative DRE promoter luciferase activity in WT and *Jdp2*^*–/–*^ MEFs in response to 10 nM TCDD for indicated time-periods. DRE luciferase activity in WT MEFs at 0 h was set at 1.0. Values represent the mean ± SEM (*n* = 3). Data were analyzed using one-way ANOVA with the Tukey post hoc test (*p < 0.05; **p < 0.01). **E** Relative ARE promoter luciferase activity in WT and *Jdp2*^*–/–*^ MEFs in response to 10 nM TCDD for indicated time-periods. ARE luciferase activity in WT MEFs at 0 h was set at 1.0. Values represent the mean ± SEM (*n* = 3). Data were analyzed using one-way ANOVA with the Tukey post hoc test (*p < 0.05; **p < 0.01). **F** Relative activity of *pGL4.1*-*AhR* luciferase in WT and *Jdp2*.^*–/–*^ MEFs treated with 10 nM TCDD for indicated time-periods. Luciferase activity was calculated as the ratio of the *AhR* luciferase activity to that of the control *pGL4.1* and is expressed as relative luciferase activity. Values represent the mean ± SEM (*n* = 5). Data were analyzed using two-way ANOVA with the Bonferroni post hoc test (*p < 0.05; **p < 0.01)

Because Jdp2 and Nrf2 play a role in the transcriptional regulation of *Cyp1b1* expression, we next investigated AhR protein expression in WT MEFs and *Jdp2*^*–/–*^ MEFs (Fig. 1A). Expression of AhR protein was significantly lower by 15–20% in *Jdp2*^*–/–*^ MEFs than in WT MEFs. This decrease in the expression of AhR protein was rescued in *Jdp2*^*–/–*^ MEFs by the forced expression of WT *Jdp2* and an alanine mutant of *Jdp2* at position 139 (Fig. 1B). To confirm that the expression of AhR is regulated by Jdp2, we treated MEFs with TCDD to stimulate AhR binding to the DRE [43]. A study of the time course of AhR expression showed increased expression of the AhR at 60 and 120 min in both WT MEFs and *Jdp2*^*–/–*^ MEFs, although the expression of AhR was twofold higher in WT MEFs than in *Jdp2*^*–/–*^ MEFs at 60 and 120 min after exposure to TCDD (Fig. 1C).

Treatment with 10 μM MG132, a proteasome inhibitor, changed the expression pattern of the AhR protein. In WT MEFs, the maximum expression of AhR protein was observed at 120 min after TCDD induction in the absence of MG132 but at 30‒60 min in the presence of MG132. However, in these cells, the expression level of AhR protein did not differ significantly in the presence or absence of MG132 (Supplementary Figure S2A). By contrast, in *Jdp2*^*–/–*^ MEFs exposed to TCDD, the expression pattern of AhR protein (Fig. 1C) and the expression level of AhR protein were similar both with and without MG132 (Supplementary Figure S2B). The expression of AhR protein was higher in WT MEFs than in *Jdp2*^*–/–*^ MEFs (Fig. 1C and Supplementary Figure S2A and B). These findings suggest that the effects of MG132 on the response of AhR protein level to TCDD did not differ significantly between WT MEFs and *Jdp2*^*–/–*^MEFs. However, the expression patterns of AhR protein in response to TCDD with and without MG132 clearly differed between WT MEFs and *Jdp2*^*–/–*^ MEFs (Supplementary Figure S2A and B). This finding suggests that Jdp2 does not alter the expression of AhR protein significantly through protease degradation. Instead, MG132 may modulate the stability of AhR protein in WT MEFs, but Jdp2 does not seem to affect the stability of AhR protein. Therefore, we first focused on the transcription of *AhR*.

### ARE, DRE, and AhR luciferase activities differ according to time after exposure to TCDD

Because the mouse *AhR* promoter region contains the ARE and DRE sequences, we first examined ARE and DRE luciferase activities in response to TCDD stimulation in WT MEFs and *Jdp2*^*–/–*^ MEFs. At 2–6 h after exposure to TCDD, DRE luciferase activity was sixfold higher in WT MEFs than in *Jdp2*^*–/–*^ MEFs (Fig. 1D). At 6–24 h after TCDD exposure, ARE luciferase activity was 5–sixfold higher in WT MEFs than in *Jdp2*^*–/–*^ MEFs (Fig. 1E). These observations are consistent with those of earlier reports [45, 46].

The response of phase I metabolizing enzymes occurred earlier, and the response of phase II metabolizing enzyme followed phase I signaling; this pattern is part of the general response involved in detoxification and antioxidation [45, 46]. For both ARE and DRE luciferases, *Jdp2*^*–/–*^ MEFs had a significantly lower activity than WT MEFs with or without TCDD. This finding suggests that Jdp2 is required to increase the activities of both systems (Fig. 1D, E).

**Fig. 2:**
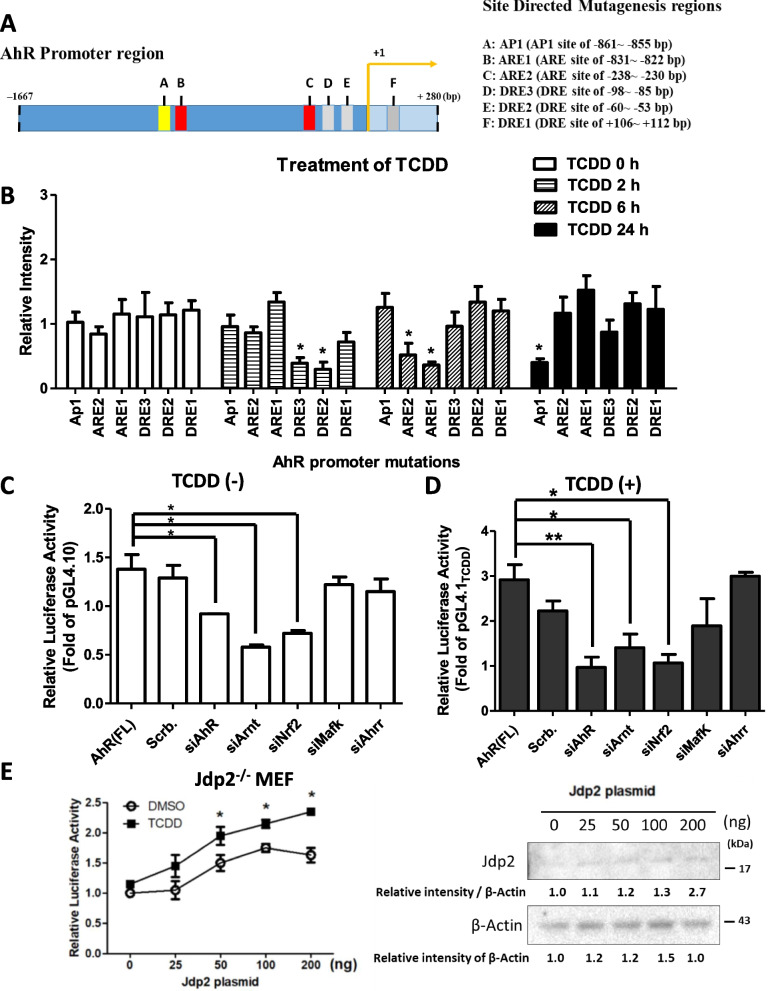
Characterization of the *AhR* promoter activity and transcription factors required for the full promoter. **A** Schematic representation of the positions of each DRE and ARE in the *AhR* promoter region. Each element is shown at the position from the putative transcription start sites, which were mutated to generate DRE and ARE mutants. **B** Effects of the mutation of each *cis*-element, ARE1, ARE2, DRE1, DRE2, and DRE3, on the *AhR* promoter region. Luciferase activity was measured in WT MEFs in the presence of 10 nM TCDD at indicated time-periods, as described in the “Methods” section. The luciferase activity of *pGL4.1*-*AhR* luciferase at 0, 2, 6, and 24 h was arbitrarily set at 1.0. Data were analyzed using one-way ANOVA with the Tukey post hoc test (*p < 0.05, *n* = 5). **C**, **D** Effects of siRNA against each representative transcription factor (*AhR*, *Arnt*, *Nrf2*, *MafK*, *Jdp2*, and *Ahrr*) on *pGL4.1*-*AhR* luciferase activity in WT MEFs in the absence (**C**) or presence (**D**) of 10 nM TCDD. The luciferase activity of *pGL4.1* luciferase was arbitrarily set at 1.0. Data were analyzed using one-way ANOVA with the Tukey post hoc test (*p < 0.05; **p < 0.01, *n* = 3). **E** Effects of the dose of the Jdp2 expression vector (0, 25, 50, 100, or 200 ng) with 10 nM TCDD treatment in *Jdp2*.^*–/–*^ MEFs. DMSO was added at 0.1%. The expression levels of Jdp2 and β-actin are shown in cropped figures. See Supplementary Figure S8 for the original full-length blot images. The intensity of each band was then quantified, and the relative value was normalized to β-actin and is shown as a ratio. Data were analyzed using one-way ANOVA with the Tukey post hoc test (*p < 0.05, *n* = 3)

To shed light on the transcriptional regulation of the AhR, the full-length construct (1,947 base pairs of the *AhR* promoter was used in the following experiments because it contains ARE, DRE, and AP-1 elements. To characterize the TCDD-mediated AhR response further, we evaluated the time course of the response of the *AhR* promoter activity to TCDD exposure (Figs. 1F and 2A). A twofold difference in *AhR* promoter activity between WT and *Jdp2*^*‒*/‒^ MEFs became evident at 2–6 h and continued to 24 h after TCDD exposure. In general, *AhR* promoter activity was significantly lower in *Jdp2*^*‒*/‒^ MEFs than in WT MEFs.

We also examined the critical role of Jdp2 in *AhR* promoter activation. Constructed recombinant short hairpin RNA (shRNA)–*Jdp2* target sites were obtained from Merck & Co., Inc. (Darmstadt, Germany) (Supplementary Figure S2C). Four shRNA segments from five vectors induced a significant reduction in AhR reporter activity. In addition, in *Jdp2*^*‒*/‒^ MEFs, the forced expression of pcDNA3–*Jdp2* increased *AhR* promoter reporter activity in the presence or absence of TCDD (Supplementary Figure S2D). We conclude that *Jdp2* plays a critical role in *AhR* promoter reporter activity.

We next performed experiments to confirm *AhR* promoter activity, which is activated by phase I but not by phase II ligands. The phase I enzyme ligands, 6-formylindolo [3,2-*b*] carbazoleand benzo[*a*]pyrene (BaP), and the endogenous phase I ligand, kynurenine, increased *AhR* promoter activity, but depletion of Jdp2 repressed *AhR* promoter activity by 50–60% in *Jdp2*^*‒*/‒^ MEFs compared with WT MEFs (Supplementary Figure S3A). Similar results were obtained in other cells such as HeLa and 293T cells (data not shown).

Next, we tested various phase II ligands such as l-sulforaphane, *tert*-butylhydroquinone (tBHQ), cinnamaldehyde, and perillaldehyde [18, 19, 22, 39, 47] (Supplementary Figure S3B–E). Perillaldehyde induced the Nrf2-related responses without activation of the AhR [16]. In addition, cinnamaldehyde inhibited AhR signaling and induced Nrf2-mediated antioxidative activity in human keratinocytes [48]. We did not detect significant induction of AhR luciferase activity in response to most phase II reagents except for tBHQ in WT MEFs (Supplementary Figure S3B–E). By contrast, in the absence of Jdp2, the *AhR* promoter was activated significantly by exposure to all examined phase II reagents. This finding suggests that Jdp2 functions as a negative regulator of the *AhR* promoter in WT MEFs. We previously reported that Jdp2 is involved in the antioxidative response by reducing ROS production in MEFs [29]. Thus, it appears that, in the absence of Jdp2, the production of ROS increases to its highest level, which then stimulates *AhR* promoter activity. These findings suggest that Jdp2 plays a critical role in repression of the AhR response followed by stimulation of Nrf2-dependent phase II enzyme reactions.

### cis-elements in the AhR promoter play a role in the response to TCDD

To investigate the role of *cis-*elements in the *AhR* promoter, which is upregulated by TCDD exposure, we performed site-directed mutagenesis experiments to replace each individual site of the DRE and ARE on the *AhR* promoter with an irrelevant DNA sequence (Fig. 2A). After transfection, WT MEFs were exposed to TCDD for 2 h (DRE), 6 h (ARE), or 24 h (AP-1 elements), and the time course of the responses was assessed. After the 2 h incubation with TCDD, DRE2, and DRE3, but not DRE1, were active. After the 6 h incubation with TCDD, ARE1 and ARE2 were active. After the 24 h incubation, only the AP-1 element was crucial for activation of the *AhR* promoter (Fig. 2B). In a further experiment, exposure to another phase I toxicant, such as BaP, resulted in similar activation of CRE2/3 site recruitment, in which the AhR, Jdp2, Nrf2, and Arnt are involved (data not shown). These findings suggest that the role of TCDD in *AhR* promoter activation is dependent on exposure to TCDD, that AhR activation is time dependent, and that the sequence of the response to TCDD involves activation of the DRE followed by the ARE and then AP-1. Jdp2 and Nrf2 seem to be involved in the regulation of *AhR* promoter activity.

To identify the molecules that bind to the ARE and DRE, we used siRNA against *AhR*, *Arnt*, *Ahrr*, and *Nfe2l2* in experiments and verified the knockdown of each gene product (Supplementary Figure S3F) [49]. siRNAs against *AhR*, *Arnt*, and *Nfe2l2* significantly repressed *AhR* promoter activity in the absence (Fig. 2C) or presence of TCDD (Fig. 2D). However, siRNAs against *MafK* and *Ahrr* did not decrease *AhR* promoter activity. As described above, both ARE1 and ARE2 *cis*-elements appear to be crucial for *AhR* promoter activation in response to TCDD (Fig. 2B).

The roles of Jdp2 and Nrf2 in *AhR* promoter activation were examined in *Jdp2*^*–/–*^ MEFs. To confirm that Jdp2 and Nrf2 are involved in the regulation of *AhR* promoter activity, pcDNA-*Jdp2* or pcDNA-*Nrf2* was transfected with the luciferase plasmids. Increasing the dose of *Jdp2* from 50 to 200 ng increased the *AhR* promoter activity by 1.9‒2.5-fold in the presence of TCDD. In cells incubated with dimethyl sulfoxide (DMSO), Jdp2 expression increased the *AhR* promoter activity by 1.4‒1.6-fold (*p* < 0.05) (Fig. 2E). Thus, the addition of TCDD upregulated the *AhR* promoter activity more than that induced by DMSO.

In these experiments, AhR autoregulated the *AhR* promoter positively in WT MEFs or negatively in the absence of Jdp2 expression. These findings suggest that AhR itself seems to be required for activation of the *AhR* promoter (Supplementary Figure S3G).

### The DRE and ARE important cis-elements in the AhR promoter

**Fig. 3:**
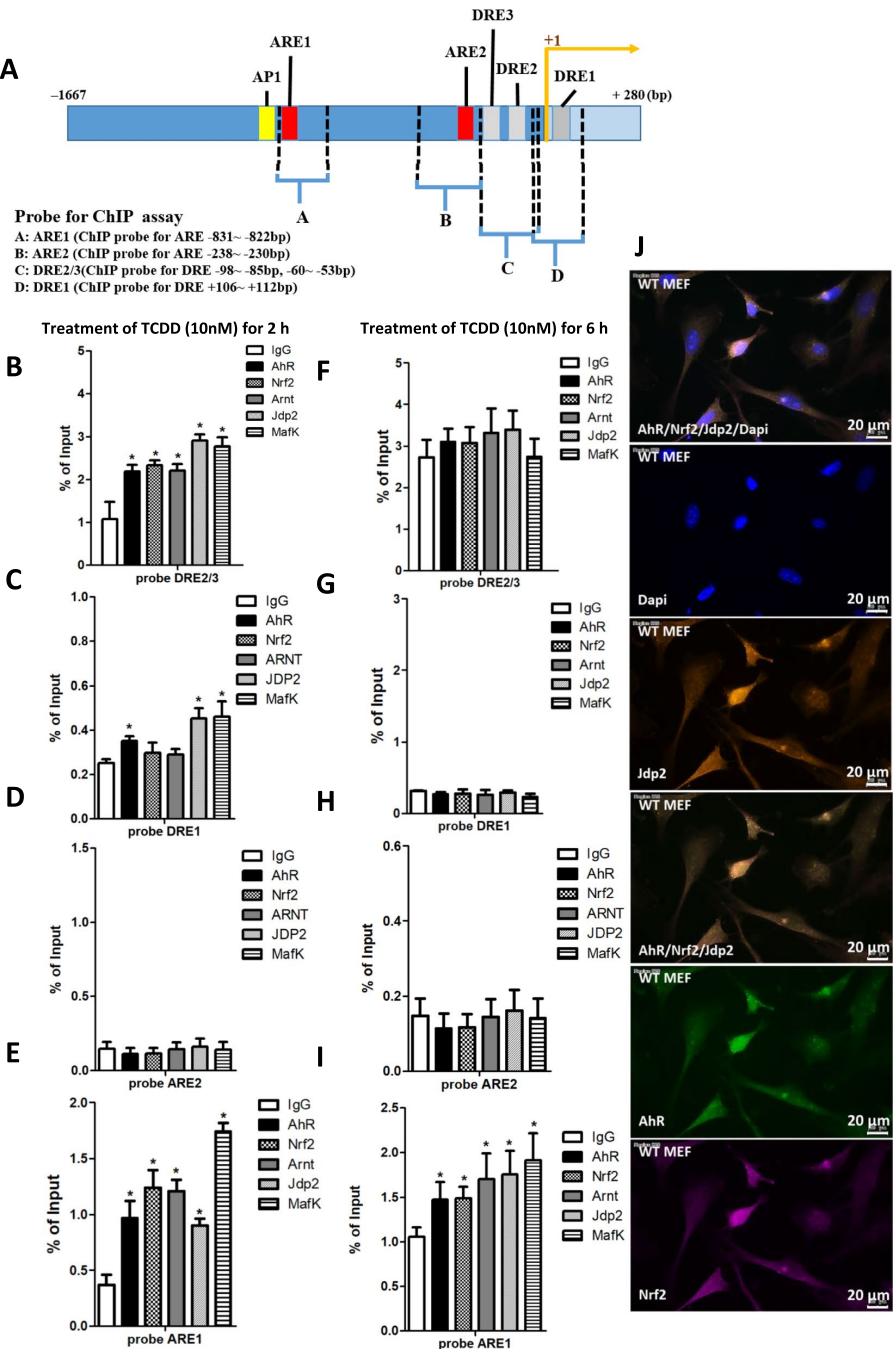
Chromatin immunoprecipitation (ChIP) assay and colocalization study of the AhR–Jdp2–Nrf2 axis. **A** Schematic representation of the mouse *AhR* promoter and the position of *cis*-elements, such as ARE1, ARE2, DRE1, and DRE2/3, which were detected using the ChIP assay. **B**–**I** Regions amplified by PCR with the specific corresponding primers (ARE1, ARE2, and DRE1) and with the primers that contained the DRE2 and DRE3 *cis*-elements, as indicated in WT MEFs. ChIP–qPCR analyses were performed using chromatin extracts from WT MEFs stimulated with TCDD for 2 h **B**–**E** or 6 h **F**–**I** using the antibodies indicated and normal IgG as a negative control. The probes for ARE1 (**E**, **I**), ARE2 (**D**, **E**), DRE1 (**C**, **G**), and DRE2/3 (**B**, **F**) are shown in the presence of 10 nM TCDD. Values represent the mean ± SEM (*n* = 5). Data were analyzed using one-way ANOVA with the Tukey post hoc test (*p < 0.05). **J** Colocalization of Jdp2, Nrf2, and AhR. WT MEFs were stained with rabbit anti-Jdp2, anti-mouse AhR (Clone A-3; Santa Cruz Biotechnology Inc., Dallas, TX, USA), anti-mouse Nrf2 (Santa Cruz Biotechnology), goat anti-rabbit IgG Alexa Fluor 594 (Themo Fisher Scientific, Walsum, MA, USA), goat anti-mouse IgG Alexa Fluor 488 (Thermo Fisher Scientific), and goat anti-mouse IgG Alexa Fluor 647 (Thermo Fisher Scientific). Scale bars, 30 μm

Chromatin immunoprecipitation (ChIP) was performed to confirm the interactions of AhR, Arnt, Jdp2, MafK, and Nrf2 with DRE and ARE *cis*-elements in the *AhR* promoter (Fig. 3A). We designed primer pairs for the ChIP assay to cover each *cis-*element, but we covered DRE2/3 with one primer C because the distance between DRE2 and DRE3 was too narrow to separate them and obtain separate PCR bands.

MEFs were treated with 10 nM TCDD for 2 or 6 h to activate the AhR–Arnt complex. After the 2 h exposure, all complexes that included the AhR, Arnt, Nrf2, Jdp2, and MafK were recruited to DRE2/3 (Fig. 3B), but only the AhR, Jdp2, and MafK were recruited to DRE1 (Fig. 3C). This DRE1 site was not functional after the 2 h treatment with TCDD, as described above (Fig. 2A, B). After the 2 h treatment with TCDD, the ARE1 and ARE2 elements were not functional (Fig. 3D, E). However, after the 6 h treatment, Nrf2 complexes involving Nrf2, MafK, Jdp2, and Arnt without the AhR had formed on ARE1 (Fig. 3I) but not on ARE2, DRE1, or DRE2/3 (Fig. 3F–H). These findings suggest that ARE complexes involving Nrf2 plus MafK in addition to Jdp2 were critical for the DRE-dependent activation during the 2 h TCDD treatment and that the sequence-specific recruitment may require AhR–Arnt and Jdp2. Thus, the early activation of AhR phase I promoter genes by phase I ligands may be controlled first through their respective DRE *cis*-elements. This is followed by ARE as recruited by the AhR–Arnt phase I complex [4] and the Nrf2–Jdp2–MafK phase II complex [29].

To confirm these observations, an immunocolocalization assay was performed using antibodies specific to AhR, Nrf2, and Jdp2. Signals for Jdp2, Nrf2, and AhR were detected in all regions of the nucleus (Fig. 3J). Most signals for Nrf2 and the AhR colocalized with those of Jdp2. Thus, the complexes encompassing the AhR, Nrf2, and Jdp2 seem to be present in the nuclei of WT MEFs, even in the absence of TCDD. In the control images, no signals were detected (data not shown).

### The AhR interacts with Jdp2 and Nrf2 in the nucleus

We next investigated whether the AhR associates with Jdp2 and Nrf2 within the cell. Endogenous proteins were coimmunoprecipitated using highly specific antibodies. Instead of using a transfection system with forced expression, we precipitated endogenous AhR directly and then performed Western blotting with high-quality antibodies to identify the interactions between molecules and the AhR in WT and *Jdp2*^*–/–*^ MEFs. Immunoprecipitation with antibody to the AhR also precipitated detectable amounts of Nrf2 in DMSO- and TCDD-treated cells, but this was detected only in the nucleus (Supplementary Figure S4A–D). Expression of nuclear AhR-associated Nrf2 after the 2 h exposure was higher in DMSO-only treated WT MEFs than in those treated with both DMSO and TCDD. By contrast, in *Jdp2*^*–/–*^ MEFs, expression of nuclear AhR-associated Nrf2 at 2 h was lower in MEFs treated with DMSO than in those treated with TCDD. These findings indicate that, in the absence of Jdp2, the Nrf2–AhR interaction was stronger after TCDD treatment than after DMSO treatment (Supplementary Figure S4A).

As a positive control, the interaction between the AhR and Arnt was examined. TCDD induced the nuclear accumulation of AhR and Arnt proteins after exposure for 2 h and 6 h in WT and *Jdp2*^*–/–*^ MEFs (Supplementary Figure S4B and C). These results suggest that, in addition to its role in the AhR–Arnt interaction, Nrf2 is also a component of the AhR complex. Nrf2 was associated with the AhR after 2 and 6 h of TCDD treatment in WT MEFs, but no AhR–Nrf2 complexes were observed after 24 h of treatment (Supplementary Figure S14B), probably because of degradation of endogenous AhR [50]. The AhR–Nrf2 complex accumulated in the nucleus after the 2 h TCDD treatment in *Jdp2*^*–/–*^ MEFs. However, no AhR–Nrf2 complex was detected at 6 and 24 h after TCDD treatment, although the AhR–AhR homodimer was present after 24 h of TCDD treatment (Supplementary Figure S4B, C).

The maximum AhR–Arnt interaction was detected after 6 h of TCDD treatment in WT MEFs. In *Jdp2*^*–/–*^ MEFs, TCDD treatment for 2 or 6 h produced the maximum response. In WT MEFs, Jdp2 interacted with Nrf2 after TCDD treatment for 2, 6, or 24 h, and the interaction was maximum after 6 h. By contrast, the interaction of Jdp2 with the AhR was detected after 2 and 6 h of TCDD treatment but not after 24 h (Supplementary Figure S14D). These findings suggest that TCDD induces multiprotein complexes to mediate the cross-interaction between the AhR–Arnt and Nrf2 pathways. Importantly, Jdp2 seems to interact with both the AhR and Nrf2 in the nucleus.

The effects of TCDD exposure on the AhR protein level were analyzed by Western blot analysis of total cell lysates. Lower AhR expression was observed in *Jdp2*^*–/–*^ MEFs than in their WT counterpart under the resting or vehicle-treated conditions (Supplementary Figure S4A). Incubation with TCDD for 6 h markedly decreased the AhR protein level in both WT and *Jdp2*^*–/–*^ MEFs (Supplementary Figure S4B, C), which was consistent with the ligand-activated AhR degradation reported previously [51]. To confirm the Western blotting results, we examined the time course of the effects of TCDD on the subcellular distribution of the AhR in WT MEFs. TCDD treatment triggered AhR nuclear translocation as early as 30 min, and this effect was reduced after 2 h but lasted for 24 h (Supplementary Figure S4E–G).

### DRE2 and 3 sites are crucial for the TCDD-dependent AhR promoter activation by Jdp2

We next examined the effects of the ectopic expression of Jdp2 and Nrf2 on TCDD-induced *AhR* promoter activity in DRE2 site mutants. In *Jdp2*^*–/–*^ and WT MEFs, an increased concentration of Jdp2 significantly increased the *AhR* promoter activity, but this effect was abolished in the presence of TCDD in both DRE2 mutants (Supplementary Figure S5A, B). These findings indicate that the DRE2 site is critical for Nrf2-mediated activation of the *AhR* promoter. However, it is unclear whether Nrf2 recognizes the DRE sequences by direct binding or whether complexes such as AhR–Arnt, Nrf2–MafK, and Jdp2 recognize DRE sequences. It is also unclear how the AhR protein recognizes the ARE sequence.

To understand these processes further, we performed an in vitro electrophoresis migration shift assay (EMSA) assay to detect GST–AhR and GST–Nrf2 proteins in the ARE and DRE *cis*-elements, respectively (Supplementary Figure S6A, B). The EMSA assay revealed that single GST–Nrf2 recombinant proteins bound the DRE2 and 3 sites and that GST–AhR basic helix–loop–helix (bHLH) recombinant proteins bound the ARE1 site. In the presence of GST–Nrf2, the binding of GST–AhR bHLH to DRE3 seemed to be increased (Supplementary Figure S6C). In addition, the GST–Jdp2 protein bound to both DRE and ARE sites, probably through the CG-rich sequences [52]. Further studies are needed to examine the critical residues that bind each *cis*-element in chromatin in complexes such as the AhR–Jdp2 and Nrf2–Jdp2 complexes [29].

### Jdp2 deficiency increases TCDD-induced ROS without AhR expression in MEFs

**Fig. 4:**
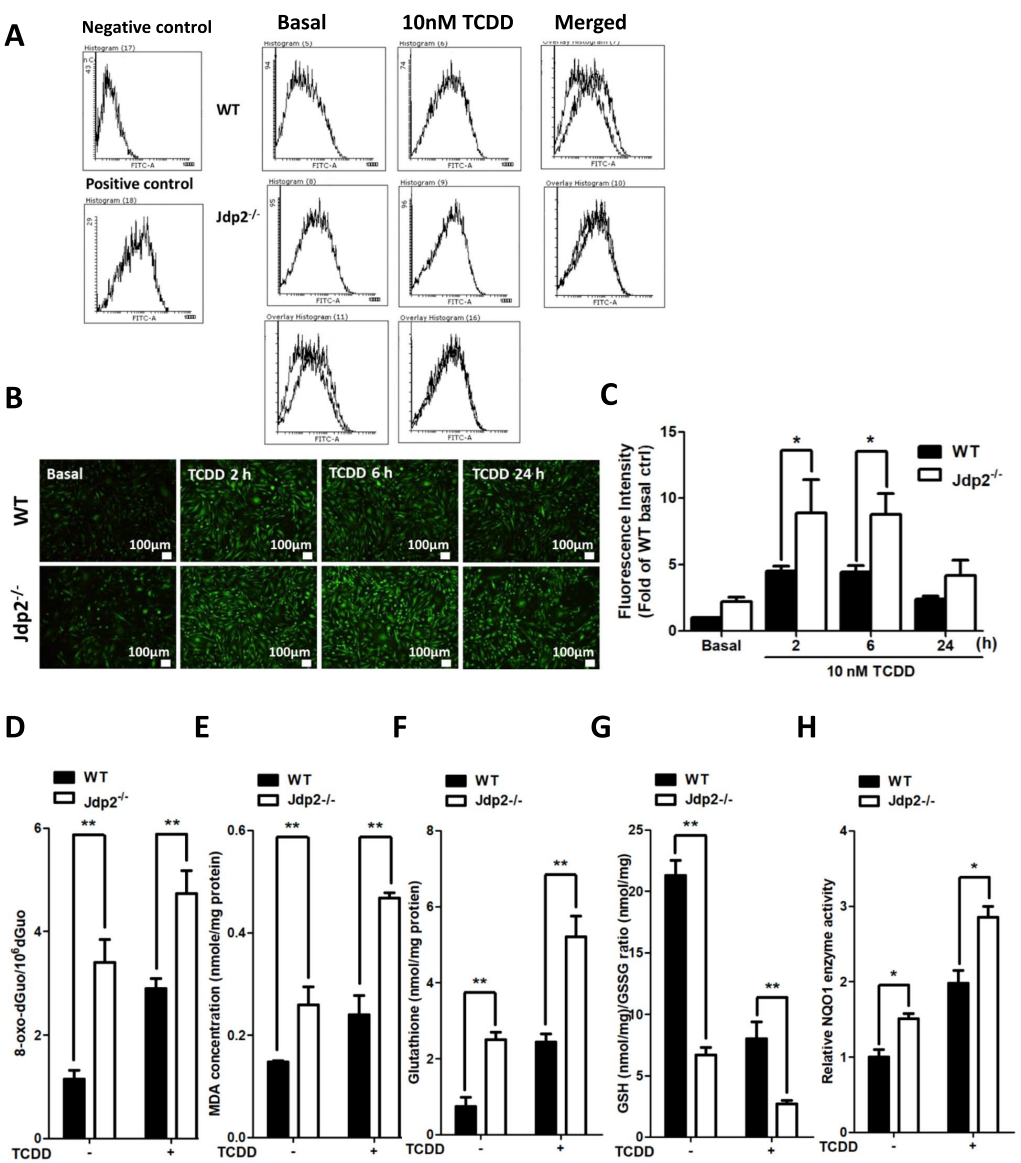
ROS activity in response to TCDD in WT and *Jdp2*^−/−^ MEFs. **A** MEFs were incubated with 10 nM TCDD for 2 h, stained with 0.25 M CM-H_2_-DCFDA, and examined by flow cytometry, as described in the “Methods” section. **B** ROS activity was measured in WT and *Jdp2*^−/−^ MEFs in response to exposure to 10 nM TCDD for 0, 2, 6, or 24 h. ROS production was detected using CM-H_2_DCFDA, as described in the “Methods” section. Representative fluorescence images of ROS generation in WT (top) and *Jdp2*^−/−^ (bottom) MEFs are shown. **C** The data obtained in the fluorescence images of ROS levels detected using CM-H_2_DCFDA after treatment with TCDD were analyzed using ImageJ software. The fluorescence intensity of WT MEFs and *Jdp2*^−/−^ MEFs after TCDD exposure was set at 1.0. Values represent the mean ± SEM (*n* = 5). Data were analyzed using two-way ANOVA with the Bonferroni post hoc test (*p < 0.05). **D**–**H** Jdp2 controls ROS production and antioxidation reaction in MEFs. **D** Levels of 8-oxo-dGuo in WT and *Jdp2*^−/−^ MEFs in the presence or absence of TCDD (10 nM) for 2 h in cells harvested at 24 h. **E** Levels of malondialdehyde (MDA) in in WT and *Jdp2*^−/−^ MEFs in the presence or absence of TCDD (10 nM) for 2 h in cells harvested at 24 h. **F**,** G** Levels of total glutathione (GSH) (**F**) and the GSH/oxidized glutathione (GSSG) ratio (**G**) in WT and *Jdp2*^−/−^ MEFs with or without exposure to TCDD (10 nM) for 2 h in cells harvested at 24 h. **H** Relative NQO1 enzyme activity in WT and *Jdp2*.^*−/−*^ MEFs with or without exposure to TCDD (10 nM) for 2 h in cells harvested at 24 h. Data are presented as the mean ± SEM (*n* = 3). Data were analyzed using one-way ANOVA with the Turkey post hoc test (*p < 0.05 and **p < 0.01)

TCDD is generally considered to induce robust ROS generation in keratinocytes via AhR activation [22, 53]. To understand the role of Jdp2 in ROS production in WT MEFs, we used chloromethyl-2′,7′-dichlorofluorescein diacetate and flow cytometry to measure ROS production, as described previously [38]. Under the steady state, ROS generation was higher in *Jdp2*^*–/–*^ MEFs than in WT MEFs (Fig. 4A, B). TCDD-evoked ROS production was maximal after 2 h and was sustained for 6 h but then declined after 24 h in both WT and *Jdp2*^*–/–*^ MEFs (Fig. 4C). However, TCDD stimulated more ROS production in *Jdp2*^*–/–*^ MEFs than in WT MEFs, which suggests that *Jdp2*^*–/–*^ MEFs exhibited greater oxidative stress. This result agrees with our earlier finding that Jdp2 is required to control the balance of oxidation and antioxidation [29]. However, the 2 and 6 h TCDD treatments were performed after cultivation for 22 and 18 h, respectively, in Dulbecco’s modified Eagle medium, which can also induce replication stress. We, therefore, chose to measure ROS production after a 2-h culture in the presence of TCDD.

We examined the production of other oxidative stress markers and redox control markers. Another marker of oxidative stress, 7,8-dihydro-8-oxo-27-deoxyguanosine, is one of the major products of DNA oxidation. Its level was higher in *Jdp2*^*–/–*^ MEFs than in WT MEFs with or without the 2 h TCDD exposure (Fig. 4D). The production of malondialdehyde (MDA) was used as an indicator of lipid peroxidation by H_2_O_2_. The MDA level was higher in *Jdp2*^*–/–*^ MEFs than in WT MEFs in the absence or presence of TCDD (Fig. 4E). These findings indicate that oxidative stress was increased in *Jdp2*^*–/–*^ MEFs and suggest that this may reflect the lower ARE activity in *Jdp2*^*–/–*^ MEFs than in WT MEFs after the 2 h TCDD exposure. The total glutathione (GSH) level was two- and threefold higher in WT MEFs than in *Jdp2*^*–/–*^ MEFs in the absence and presence of TCDD, respectively, and TCDD decreased the glutathione levels by 33% to 50% (Fig. 4F). In *Jdp2*^*–/–*^ MEFs, the GSH/oxidized glutathione, ratio was decreased by 31.4% and 33.7% in the absence or presence of TCDD, respectively (Fig. 4G). This decrease may have contributed to a decrease in oxidative stress in *Jdp2*^*–/–*^ MEFs. NQO1 enzyme activity was 1.4- and 1.5-fold higher in the presence or absence of TCDD, respectively, in *Jdp2*^*–/–*^ MEFs than in WT MEFs (Fig. 4H). These results were coincident with the changes in the ROS production level (Fig. 4A–C).

### **AhR target genes are expressed differently in WT and Jdp2**^**–/–**^** MEFs**

qPCR was used to examine the effects of TCDD incubation on the expression of the genes aldehyde dehydrogenase 3 family member A1 (*Aldh3a1*), *Cyp1b1*, TCDD-inducible poly(ADP-ribose) polymerase (*Tiparp*), and the AhR repressor (*Ahrr*) (Supplementary Figures S1B and 7A–C). The mRNA expression levels of these genes under DMSO treatment were similar in WT and *Jdp2*^*–/–*^ MEFs. However, incubation with 10 nM TCDD for 2 h markedly increased *Aldh3a1*, *Cyp1b1*, and *Tiparp* mRNA expression in WT MEFs. By contrast, the expression levels of *Aldh3a*, *Cyp1b1*, and *Tiparp* were lower in in *Jdp2*^*–/–*^ MEFs than WT MEFs. These results suggest that Jdp2 is involved in AhR signaling to its downstream target genes in a ligand-dependent manner.

### Jdp2 deficiency alters cytoskeleton remodeling and cell spreading but has limited effects on myosin light chain phosphorylation

**Fig. 5:**
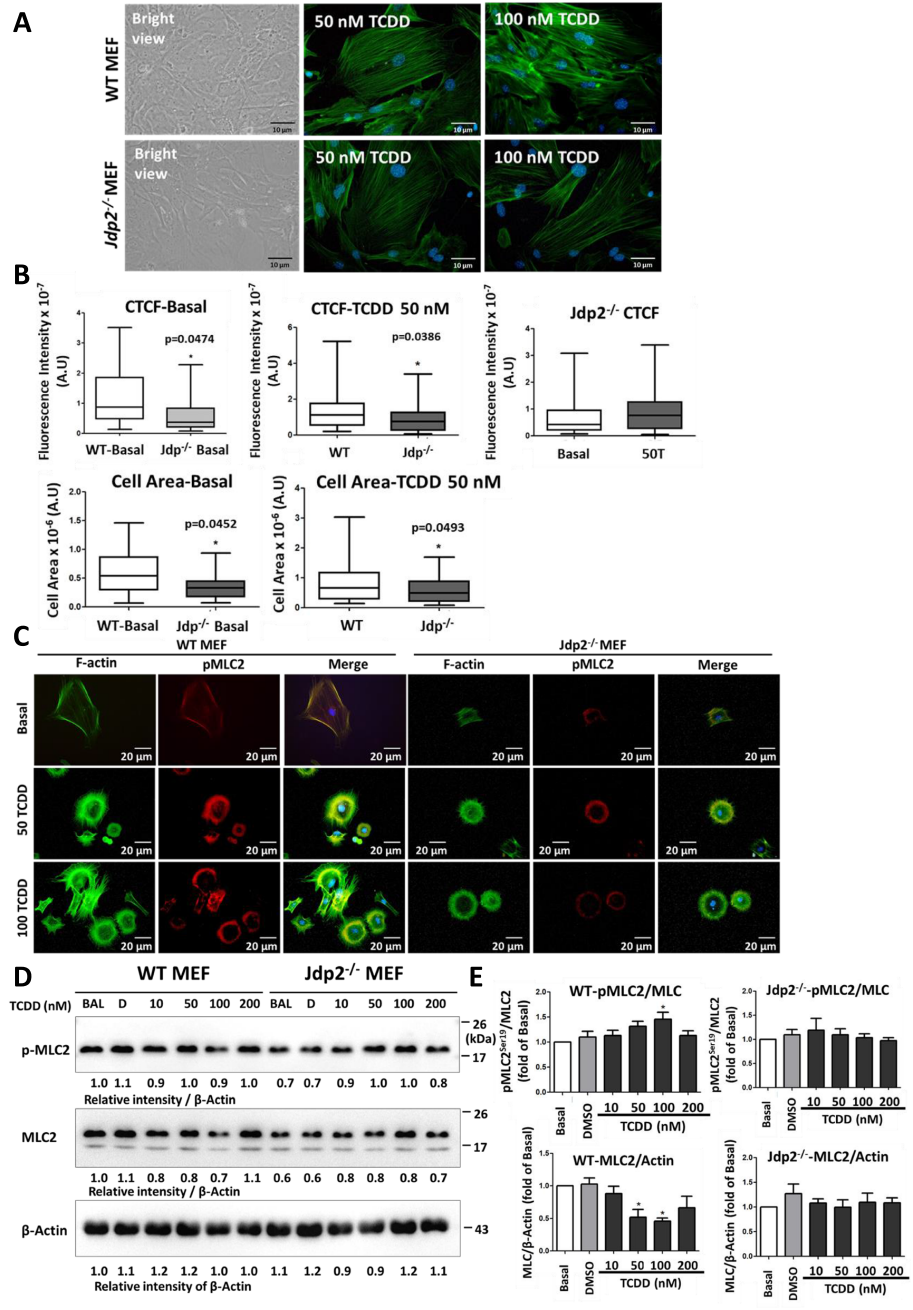
Attenuation of TCDD-induced spreading of actin stress fibers by *Jdp2* deficiency. **A** Cells were serum starved overnight and then treated with DMSO and TCDD for 24. At the end of treatment, the cells were rinsed with PBS, fixed with 4% formaldehyde, and processed for F-actin staining with Alexa 488-conjugated phalloidin. Nuclei stained blue with DAPI. For each treatment, at least five fields were acquired. **B** The fluorescence intensity was measured in WT and *Jdp2*^*–/–*^ MEFs in the presence or absence of 50 nM TCDD. CCCTC binding factor CTCF and cell area were measured as described in the “Methods” section. **C** Extension of cell spreading of WT and *Jdp2*^*−/−*^ MEFs after exposure to TCDD, and p-MLC2 and F-actin expression upon TCDD challenge in WT and *Jdp2*^*−/−*^ MEFs. Representative staining of F-actin and p-MLC2, and merged images are shown. **D**, **E** Western blot analysis of p-MLC2 and total MLC2 expression (**D**) along with the quantitative results (**E**). Cropped figures are shown. See Supplementary Figure S18 for the original full-length blot images. The intensity of each band was then quantified. The relative value was normalized to β-Actin and shown as ratio. **E** for cells harvested at 6 h after treatment with 0–200 nM TCDD. The cell lysates were processed to measure p-MLC2 content, as described in the “Methods” section. Data are presented as mean ± SEM from three independent experiments and *p* values were obtained

In addition to its role in xenobiotic metabolism, emerging evidence also suggests that the AhR may be involved in the regulation of cell plasticity and mobility, which are associated with cytoskeleton remodeling [54]. To examine the role of Jdp2 and the functional relevance of the AhR in cytoskeleton-related events, phalloidin staining of actin and cell spreading assays were performed after TCDD treatment at concentrations of 50, 100, and 200 μM of WT and *Jdp2*^*–/–*^ MEFs for 24 h. In WT MEFs, actin stress fiber formation was higher after TCDD treatment than after DMSO treatment. TCDD increased the formation of F-actin fibers less in WT MEFs than in *Jdp2*^*–/–*^ MEFs (Fig. 5A). The cell morphology was rounder in *Jdp2*^*–/–*^ MEFs than in WT MEFs, and TCDD treatment of WT MEFs induced the loss of roundness to that seen in *Jdp2*^*–/–*^ MEFs. In the analysis of cell spreading, both the cell area and actin cytoskeleton fluorescence intensity were slightly lower in *Jdp2*^*–/–*^ MEFs than in WT MEFs treated with 50 or 100 nM TCDD (Fig. 5B, C).

Phosphorylated myosin light chain 2 (pMLC2) is a critical component of actin stress fiber remodeling. To examine the effects of TCDD on actin cytoskeleton remodeling, we performed Western blot analysis to measure pMLC2 content in WT and *Jdp2*^*–/–*^ MEFs treated with 10–200 nM TCDD for 6 h. Under the steady-state condition, Jdp2 deficiency slightly decreased MLC2 content. TCDD treatment at 100 nM increased MLC2 content, but its phosphorylation was significantly decreased by TCDD at 50 and 100 nM in WT MEFs. By contrast, MLC expression and phosphorylation levels were constant at different TCDD concentrations in *Jdp2*^*–/–*^ MEFs (Fig. 5D, E).

### The AhR is the downstream product of Jdp2-dependent signaling during cell migration and pancreatic cancer development

**Fig. 6:**
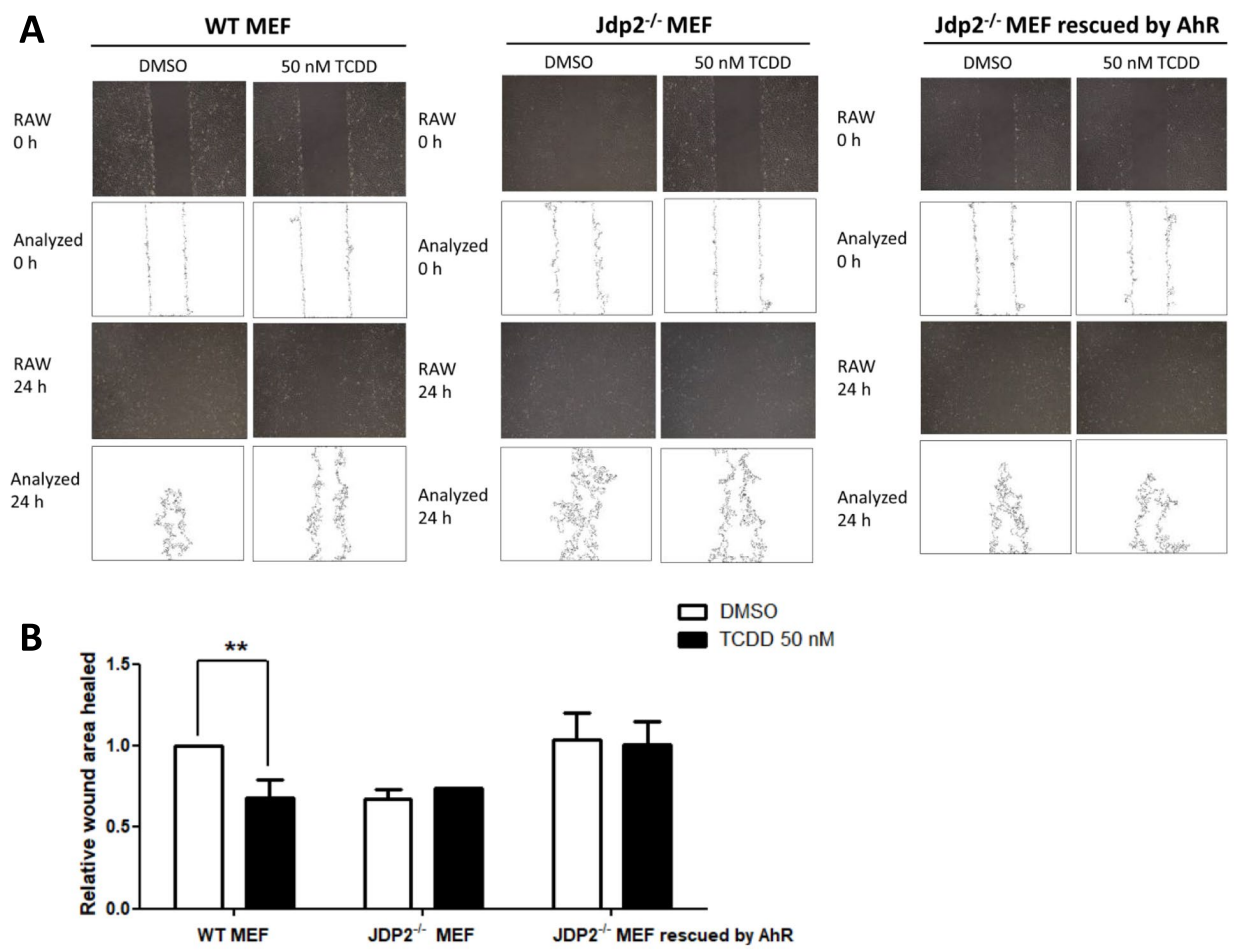
Rescue of TCDD-induced cell spreading by overexpression of AhR in MEFs. **A** Rescue after 2 h exposure to TCDD and wound healing by 50 ng of *pcDNA-HA-AhR* in *Jdp2*^−/−^ MEFs. **B** Addition of 50 ng TCDD repressed the cell spreading activity of WT MEFs by 60–70% but did not affect cell spreading of *Jdp2*.^*−/−*^ MEFs. This repression was rescued by the forced expression of AhR. The relative wound area was measured. Data represent the mean ± SEM (*n* = 5). Data were analyzed using one-way ANOVA with the Tukey post hoc test (**p < 0.01)

Because Jdp2 decreased actin stress fiber spreading, probably by dysregulating the cytoskeleton, we used a wound-healing assay to examine further whether the cell migration ability is affected by cell proliferation. We first treated cells for 24 h with 50 nM TCDD and then used ImageJ software to measure the healed area (Fig. 6A). *Jdp2*^*–/–*^ MEFs showed significantly lower migration ability than WT MEFs when incubated with DMSO or 10 nM TCDD. To determine whether Jdp2 is involved in the control of cell migration, we transfected *pcDNA-AhR* into *Jdp2*^*–/–*^ MEFs to determine whether overexpression of the AhR can rescue the migration loss in *Jdp2*^*–/–*^ MEFs. Intriguingly, 50 nM TCDD significantly decreased the migration ability of transfected WT MEFs but not of *Jdp2*^*–/–*^ MEFs (Fig. 6B).

Taken together, these findings suggest a role of Jdp2 in AhR-mediated ROS functions after exposure to phase I ligands such as TCDD. This role seems to include the transcriptional activation of Jdp2 through the recruitment of the Nrf2–MafK complex on the *AhR* promoter in a spatiotemporal manner. Jdp2 may play a critical role in the regulation of ROS through *AhR* transactivation. Functionally, Jdp2 seems to control the TCDD-induced cell spreading and migration of mouse MEFs.

**Fig. 7:**
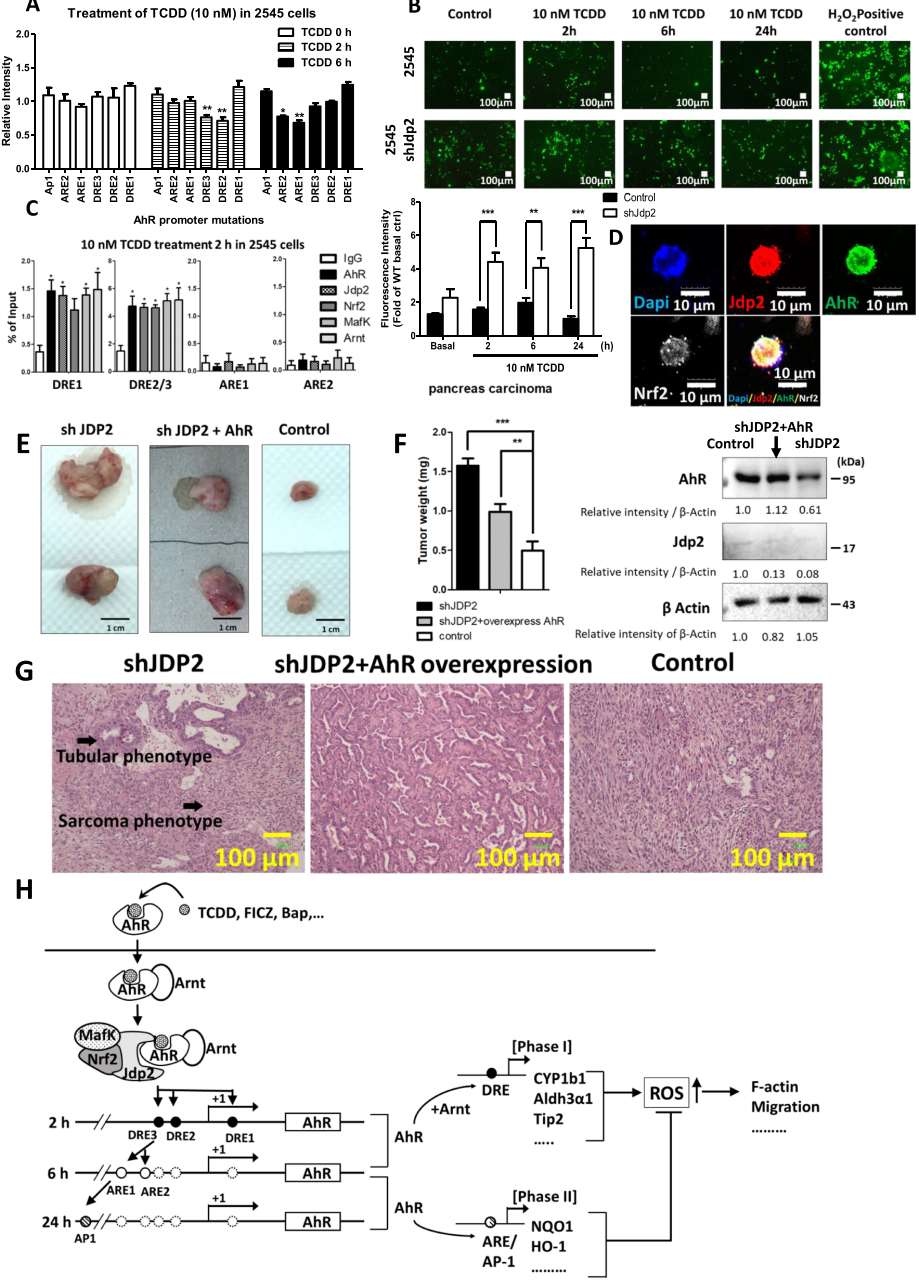
Rescue of tumorigenesis by overexpression of AhR in sh*Jdp2-*treated *Kras-Trp53*-mutated pancreatic carcinoma 2545 cells. **A**–**C** The role of the Jdp2–AhR axis in pancreatic cancer 2545 cells was examined in response to TCDD using *AhR* luciferase, ROS generation, and ChIP assays. **A** Effects of the mutation of each *cis*-element, ARE1, ARE2, DRE1, DRE2, and DRE3, on the *AhR* promoter region. Luciferase activity was measured in 2545 cells in the presence of 10 nM TCDD at indicated time-periods, as described in the “Methods” section. The luciferase activity of full-length (FL) *AhR* luciferase was arbitrarily set at 1.0. Values represent the mean ± SEM (*n* = 5). Data were analyzed using one-way ANOVA with the Tukey post hoc test (*p < 0.05). **B** ROS activity was measured in 2545 cells in response to exposure to 10 nM TCDD for indicted time-periods. ROS production was detected using CM-H_2_DCFDA, as described in the “Methods” section. Representative fluorescence images of ROS generation in 2545 cells (top) and 2545 cells treated with sh*Jdp2*-treated 2545 cells (bottom) are shown. The data obtained in the fluorescence images of ROS levels detected using CM-H_2_DCFDA after treatment with TCDD were analyzed using ImageJ software. The fluorescence intensity of 2545 cells without TCDD was set at 1.0. Values represent the mean ± SEM (*n* = 5). Data were analyzed using two-way ANOVA with the Bonferroni post hoc test (**p < 0.01; ***p < 0.001). **C** ChIP assay of the AhR–Jdp2–Nrf2 axis. Regions amplified by PCR with the specific corresponding primers (ARE1, ARE2, and DRE1) and with the primers that contained the DRE2 and DRE3 *cis*-elements as indicated in WT MEFs. ChIP–qPCR analyses were performed using chromatin extracts from 2545 cells stimulated with TCDD for 2 h with the indicated antibodies and normal IgG (as a negative control). The probes for ARE1, ARE2, DRE1, and DRE2/3 were used in the presence of 10 nM TCDD. Values represent the mean ± SEM (*n* = 5). Data were analyzed using one-way ANOVA with the Tukey post hoc test (*p < 0.05). **D** Coimmunostaining of AhR–Nrf2–Jdp2 protein complexes in 2545 tumor cells. 2545 cells were stained with rabbit anti-Jdp2 (a gift from A. Aronheim), rat anti-Nrf2 (Cell Signaling Technology), and goat anti-AhR (Santa Cruz Co.); with Alexa-Fluor® 488 conjugated Rabbit anti-Goat IgG (Thermo Fisher Scientific), Alexa Fluor® 594-labeled goat anti-rabbit IgG (Thermo Fisher Scientific), and Alexa Fluor® 647-labeled goat anti-rat IgG (H + L) (Cell Signal Technology). The cells were stained by 4′,6′-diamino-2-phenylindole (DAPI), to detect cell nuclei (Sigma-Aldrich). Scale bars, 10 μm. **E** Xenograft transplantation of mouse 2546 pancreatic cancers, *Jdp2*-knockdown cells, and AhR-forced-expressed *Jdp2*-knockdown 2546 cells was performed as described in the “Methods” section. Xenotransplantation and tumor formation assays were performed as described previously [36]. 2546 pancreatic tumor cells (1 × 10^6^ cells) and the transfectants by *CSIV-CMV-AhR-IRES2-Venus* virus were cultured for 2 days, and 1 × 10^5^ cells were inoculated into SCID mice as described previously [30]. **F** Tumor weight was calculated in three replicates. Data were analyzed using one-way ANOVA with Tukey’s post hoc multiple-comparison test. The expression of AhR and Jdp2 cropped figures are shown. See Supplementary Figure S8 for the original full-length blot images. The intensity of each band was then quantified. The relative value was normalized to β-Actin and shown as ratio. **G** Representative results for tumor biopsies stained with hematoxylin and eosin. The region labeled with an arrowhead indicates a different carcinoma phenotype. The characteristics of each treatment are shown for following phenotypes: sh*Jdp2* treatment produced a large area of necrosis, more epithelial–mesenchymal transition phenotypes, and the sarcoma phenotype; sh*Jdp2* + AhR overexpression produced a smaller area of necrosis and mainly the epithelial phenotype; the control produced results like those produced by sh*Jdp2* treatment and smaller areas of necrosis. **H** Schematic representation of DMSO-induced AhR activation through the complexes of AhR-Jdp2, Nrf2-Jdp2, and AhR-Nrf2 to increase ROS production, cell spreading, and apoptosis in WT MEFs. In *Jdp2*^−/−^ MEFs, only a residual amount of AhR‒Arnt was recruited to the DRE2 and DRE3 elements of the *AhR* promoter

To confirm this signaling, we used the *Kras-Trp53* knockout (KO) cancer cell line derived from *Kras-Trp53* double KO mice [30]. To examine whether the Jdp2–AhR signaling axis was similar in the *Kras-Trp53* cancer cell line and MEFs, we investigated the effects of Jdp2–AhR signaling on DRE2-dependent *AhR* promoter activity, DRE2/3-dependent recruitment of Jdp2 to the *AhR* promoter, and Jdp2-dependent ROS activity. The effects of DRE-mediated transactivation of Jdp2 on the *AhR* promoter were investigated in the pancreatic cancer 2545 cell line (Fig. 7A). The same level of repression was observed in DRE2 and DRE3 mutants after 2 h of TCDD-induced *AhR* promoter activation in 2545 cells. The TCDD-mediated ROS activity was also examined in 2545 cells (Fig. 7B). Exposure of 2545 cells to TCDD for 2–24 h caused ROS production, and sh*Jdp2* treatment blocked this ROS production. A ChIP assay using 2545 cells in the presence of TCDD was also performed (Fig. 7C). After the 2 h exposure to TCDD, the AhR, Jdp2, Nrf2, MafK, and Arnt were recruited to DRE2/3 sites (Fig. 7C). After the 6 h treatment, Nrf2, Jdp2, Arnt, Jdp2, and MafK were also recruited to the ARE1 site in 2545 cells (data not shown). Immunocytochemistry study showed the colocalization of AhR, Nrf2, and Jdp2 proteins in 2545 cells (Fig. 7D). After exposure to TCDD, DRE1 was not critical for *AhR* promoter activation (Fig. 7A). These results suggest that DRE2 and DRE3 are critical for TCDD-induced *AhR* promoter activation.

We next generated pancreatic cancer cells and used them in a xenograft transplantation assay. The introduction of sh*Jdp2* resulted in the enlargement of pancreatic tumors and about a 1.5-fold increase in the size of tumors. In this system, Jdp2 seemed to act as a tumor suppressor. We next induced AhR overexpression and found that it decreased the tumor weight (Fig. 7E, F) and decreased the number or percentage of cells exhibiting necrosis. We observed no formation of blood vessels or sarcoma and no epithelial–mesenchymal transition phenotypes (Fig. 7B). These findings suggest that the role of DRE2 and 3 is critical to the response of the Jdp2–AhR pathway to TCDD in 2545 cells and that this seems to be similar to that in MEFs. Taken together, these data imply that the Jdp2‒AhR cascade may be crucial for TCDD-induced *AhR* promoter activation, ROS generation, and DRE2/3-mediated recruitment to the *AhR* promoter. The AhR is downstream of Jdp2 in the signaling cascade in MEFs and *Kras-Trp53*-mutated pancreatic cancer 2545 cells.

## Discussion

ROS homeostasis is controlled by the balance of oxidant production and antioxidation reactions. The coordinated induction of the phase I and phase II enzyme families is the main driving force of ROS production and is involved in the modulation of cell signaling and death [55, 56]. The present study shows that transcription of the AhR, a key regulator of phase I enzymes, is regulated in a spatiotemporal manner by Nrf2 and small Maf, which are regulators of phase II enzymes, as well as through mediation by the *Jdp2* transcription factor [29]. Our findings suggest that both the AhR and Nrf2 batteries are essential for *AhR* gene expression and ROS production, and that Jdp2 is a linking factor for both enzyme batteries, which function to maintain ROS homeostasis.

We previously reported that Jdp2 is a stimulator of the antioxidation ARE reaction through its complex with Nrf2 and MafK [29]. Here, we also found that Jdp2 is a stimulator of the *AhR* promoter, which controls oxidative stress and ROS production. Our research addressed the question of how Jdp2 increases the activities of both phase I and phase II enzymes in cells exposed to phase I ligands. We found that the initial commitment occurs through the determination of the specificity that controls how AhR–Arnt is recruited to the DRE, after which the AhR is degraded and there is a switch from the DRE to ARE to maintain homeostasis. Our findings suggest that AhR degradation is required for *ARE* gene activation and that Jdp2 is the positive controller of the process from oxidative stress to antioxidation, which maintains ROS homeostasis.

TCDD is a potent activator of the AhR battery by binding to the ligand-binding pocket of the AhR–Heat shock protein 90– AhR-interacting protein complex [7]. The *AhR* gene battery encodes both phase I enzymes, such as CYP1A1, 1A2, and 1B1, and phase II enzymes, including NQO1, GSTA2, and UDP-glucuronosyltransferase 1–6 [37]. AhR agonists such as TCDD induce the activation of both types of enzymes. NQO1 and GSTs are also members of the Nrf2 gene battery. Yeager et al. [17] showed that Nrf2 is required for TCDD induction of the classical AhR battery genes *Nqo1* and most *Ugt* and *Gst* forms in mouse liver and that induction of both in succession is necessary, yet insufficient by themselves, for induction of drug-processing genes in vivo*.* Use of the ChIP assay in mouse liver cells has shown that the AhR binds directly to the DRE-like sequence in the 5′-flanking region of mouse *Nfe2l2* [6]. Wang et al. [15] also reported that, in addition to the AhR–Arnt interaction, TCDD stimulates interactions between the AhR and Nrf2, and between the AhR and Keap1. In the nucleus, TCDD may stimulate the formation of a complex of AhR, Arnt, and Nrf2 that binds to the DRE- and ARE-containing enhancer NQO1.

This AhR-dependent activation of Nrf2 by TCDD is consistent with the delayed activation and DNA-binding kinetics of Nrf2 compared with the kinetics of AhR activation. AhR- and Nrf2-mediated gene expression is linked to an overlapping response designated the “AhR–Nrf2 gene battery” [18]. The presence of the DRE and ARE in the *Nfe2l2* promoter implicates the involvement of AhR–Arnt recruitment in the Nrf2-mediated regulation of antioxidant defenses [6], whereas the Nrf2–small Maf complex regulates AhR expression [42]. However, the interactions between the AhR and Nrf2 in the DRE and ARE, and the involvement of the *AhR* promoter as the first hit in mediating gene expression have not been clarified. In addition, the role of secondary responses, such as AhR-elicited production of ROS and subsequent activation of Nrf2, is still unclear. Therefore, we focused on the role of crosslinking genes such as *Jdp2* in the ligand-specific activation of the *AhR* promoter and the possible cooperation with the Nrf2 complex.

We have provided several lines of evidence, as indicated below, that AhR agonists induce the activation of Jdp2 and that, once activated, Jdp2 can associate with the AhR and Nrf2 components and then recruit these complexes to the DRE in the *AhR* promoter in a spatiotemporal manner. First, Jdp2 seems to play a critical role in the activation of *AhR* (Fig. 1). In our study, after the 2 h exposure to the phase I ligand TCDD, the DRE element in the *AhR* promoter was critical for determining the specificity of phase I gene expression. After the 6 h exposure, the ARE element was important for increasing *AhR* promoter activity. After the 24 h exposure, nuclear AhR protein was degraded, and the AP-1 site was critical for maintaining *AhR* promoter activity (Fig. 2B). These findings indicate that the *AhR* promoter acts as part of the spatiotemporal machinery of the AhR battery and Nrf2 battery, which are connected to Jdp2 as histone chaperone-like molecules.

The second line of evidence is that AhR agonists activated the Jdp2-mediated AhR and Nrf2 complexes on the DRE2 element, which was followed by participation of the ARE1 element and finally the AP-1 site in the activation of the *AhR* promoter. Third, our studies show that, in response to AhR agonists, Jdp2 first acts as an upstream element or a coordinated component in the AhR battery followed by the Nrf2 battery. Fourth, we found that Jdp2 controls AhR–Nrf2-mediated ROS production in response to TCDD to maintain ROS homeostasis.

In our study, ROS production started at 2 h after TCDD exposure, was maximum at 6 h, and then decreased at 24 h. This ROS production was dependent on DRE commitment. The fourth line of evidence is our observation that Jdp2 seems to play a critical role in cell spreading and migration in response to TCDD as the initial hit. In the presence of TCDD, Jdp2–AhR, Jdp2–Nrf2, Jdp2–MafK, and AhR–Arnt were detected as a large complex that was recruited to the DRE2 element and promoted activation of the AhR after the initial hit. This process opened chromatin, which allowed the recruitment of RNA polymerase II, probably in response to the histone chaperone activity of Jdp2 [26, 27].

The fifth line of evidence that AhR agonists induce the activation of Jdp2 is our observation that Jdp2 is an upstream factor for AhR-mediated transcription for phase I enzyme family members and an Nrf2 transcription factor for phase II enzyme family members, which help to maintain ROS homeostasis. Differences in the responses to AhR agonists may lead to the differences of the activation complex involving Jdp2 or Nrf2. After the 2 h response to DMSO, WT MEFs showed greater interaction between AhR and Nrf2 in the nucleus compared with 2 h treatment with TCDD. By contrast, greater interaction between Arnt and AhR, which formed the complex, was higher after the 2 h treatment with TCDD than with DMSO. However, these differences were not detected in *Jdp2*^*−/−*^ MEF (Supplementary Figure S4A–C). These results suggest that Jdp2 is crucial for modulating the complex of the AhR-Jdp2-Nrf2 gene battery. Taken together, our findings suggest that Jdp2 is a transcription factor that controls the coordinated transcriptional activation of the *AhR* promoter in AhR–Nrf2 and then activates the AhR and Nrf2 gene batteries to maintain ROS production in MEFs in response to phase I ligands such as TCDD (Fig. 7H).

Contradictory roles for Nrf2 in cancer occurrence and development have been reported previously [47]. In *Nfe2l2*^*–/–*^ mice, a protective role of Nrf2 activation has been established for chemical- and radiation-induced tumorigenesis [49, 57]. Nrf2 prevents carcinogenesis by quenching ROS or quickly repairing oxidative damage or errors in enzymatic metabolism and the excretion of chemicals. These preventive activities have been reported elsewhere [53, 58, 59]. By contrast, Tao et al. reported that Nrf2-based chemoprevention was not effective against genetically induced oncogenic activation in a *Kras* G12D lung cancer model [60]. In the past decade, studies have shown that Nrf2 activation in cancer cells promotes cancer progression [38, 61] and metastasis [62], and confers resistance to chemo- and radiotherapy [58, 59]. Thus, Nrf2 seems to have two contradictory functions [37]. The data from our studies suggest that Nrf2 is involved in both phase I and phase II enzyme reactions, which induce both ROS-promoting and antioxidation aspects in a cell context-specific and ligand context-specific manner. This may explain the two different aspects of Nrf2 function.

Pancreatic cancer 2545 were used as a model to confirm the role of Jdp2 in the Nrf2 gene battery of *Kras-Trp53*-mutated cancer cells. In general, the exogenous and endogenous phase I ligands can induce ROS production through the AhR pathway and ROS protection via Nrf2 activation. This ROS production alters some cell function, such as cell migration, apoptosis, inflammation, and the epithelial-mesenchymal transition, which is required for cancer initiation, by disrupting the balance of cellular ROS generated by the AhR-Nrf2 gene battery. Here, we found that Jdp2 signaling in MEFs was involved in the induction of the DRE2-mediated *AhR* promoter response, colocalization of the AhR, Jdp2, and Nrf2, and ROS production (Fig. 7). Inhibition of Jdp2 expression significantly increased the growth of tumors but could be rescued by AhR overexpression, which suggested that Jdp2 acts as a tumor suppressor upstream of the AhR-Nrf2 gene battery (Fig. 7E).

We examined the response of pancreatic cancer 2545 cells to ROS, which involves the Warburg effect. The ChIP assay demonstrated that, after 2 h exposure to TCDD, AhR-Arnt, Nrf2-MafK, and Jdp2 components were recruited into ARE1 elements in WT MEFs but not in 2545 cells (Fig. 4B vs Fig. 7B) and that the ARE1 elements was not functional in the activation of the *AhR* promoter after 2 h exposure of TCDD in either cell type (Fig. 2B and 7A). One explanation is that the basal signals in the experiments with CM-H_2_DCFDA were significantly stronger in 2545 cells (Fig. 7B) than in WT MEFs (Fig. 4B). Thus, the relative sensitivity of TCDD-induced ROS production after 2 h exposure is higher in WT MEFs than in 2545 cells. These findings suggest that the relevant components were ready to be recruited faster to the ARE1 after 2 h TCDD exposure in WT MEFs but that this recruitment process might be slower in 2545 cells. However, the recruitment to the ARE1 in both WT MEFs and 2545 cells are not functional in *AhR* promoter activation after 2 h exposure of TCDD.

The TCDD-induced phosphorylation of MLC2 and the AhR that rescued cell migration activity in *Jdp2*^*–/–*^ MEFs was also dependent on Jdp2-mediated control of ROS (Figs. 5 and 6). We observed the presence of the AhR-Nrf2 axis in pancreatic cancer 2545 cells and that Jdp2 plays a crucial role in pancreatic cancer progression. The AhR rescued ability for tumorigenesis in *Jdp2* knockdown 2545 cells induced tumorigenesis (Fig. 7E–G). However, we cannot deny the presence of a time gap between the TCDD induced Jdp2 activation of cell migration and spreading, and pancreatic cancer malignancy. We believe that the commitment of ROS-dependent actions was detected as the effects on cell migration and spreading (Figs. 5 and 6) and tumorigenesis (Fig. 7). The DNA-binding mutant FL34R (114 and 121) of Jdp2 cannot associate with AhR protein, although FL34R was reported not to bind to Nrf2 and MafK protein [29]. In addition, alanine mutants of histone chaperones N91A and I47A of Jdp2 protein are not functional and do not associate with the AhR [44]. Epigenetic control is another possible way that Jdp2 regulates the AhR–Nrf2 axis. The downregulation of the long noncoding RNA AGAP2-AS1 competes for microRNA 574 at the posttranscriptional level and then inhibits the expression of endogenous Jdp2 [63]. AGAP2-AS1 appears to control epigenetic regulators such as EZH2 and LSD1 [64]. Jdp2 also regulates the expression of the Ink4a and Arf factors to control the EZH2 complex [37]. It is possible that AGAP2-AS1 controls the AhR‒Nrf2 battery through Jdp2 on the polycomb complex.

Jdp2 seems to be a mediator of the AhR and Nrf2 batteries and to be involved in the control of TCDD-induced activation of the *AhR* promoter. We suggest that phase I and phase II responses are required for the metabolism of toxic substances and detoxification, as well as the antioxidation reaction needed to maintain ROS homeostasis. The functions of the AhR in MEFs are not apoptosis, senescence, and autophagy, but rather alterations in the contractile proteins and cell migration ability. Cell migration is the initial step required for cell invasion and metastasis spread during cancer development [54]. For example, bone marrow-derived cells (BMDCs) secrete chemokines such as Ccl5 that potentiate metastasis, but *Jdp2*^*–/–*^ BMDCs do not induce invasion of Lewis lung carcinoma cells [65]. The initial hit to the Jdp2-mediated *AhR* promoter involves cell spreading and migration but does not commit the cell to apoptosis and senescence. Instead, the initial hit changes the cellular contractile system to adapt its metabolism and plasticity, which induces environmental alterations that maintain ROS homeostasis. These processes are dependent on the AhR–Nrf2 network and involve Jdp2 as a mediator. Further research is required to understand better the AhR‒Jdp2‒Nrf2 axis, which controls ROS homeostasis and activates the *AhR* promoter as the initial hit in the oxidative stress responses and antioxidation pathways.

## Conclusions

The control of the ROS through the balance between oxidative stress and antioxidation is critical for pharmacological, allergic, and immune responses, the epithelial to mesenchymal transition and its reverse, and cancer development. It is of interest that the phase II transcription factor Nrf2 is recruited to the AhR phase I promoter with its own AhR–Arnt complex and Jdp2 as the mediator. This finding is novel and surprising because phase I and II transcription factors are associated through Jdp2 mediators and are involved in the regulation of the AhR promoter activity. This study provides new information about the interactions of the AhR–Nrf2 battery via Jdp2 in normal MEFs and pancreatic cancer cell lines with mutations of *Trp53* and *Kras*. Use of small molecules for screening of AhR–Jdp2–Nrf2 interactions is one possible approach for controlling body homeostasis in response to both external and internal stressors.

### Supplementary Information

**Additional file 1: Supplementary Figure S1. **Luciferase activity driven by the *Cyp1b1 *promoter in response to TCDD. **Supplementary Figure S2.** Characteristics of AhR protein expression and effects of Jdp2 on AhR expression. **Supplementary Figure S3. **Characterization of *AhR *promoter activity. **Supplementary Figure S4.** Interaction of the AhR–Jdp2–Nrf2 axis in nuclei and cytoplasm in WT and *Jdp2–/–*MEFs in response to TCDD or DMSO exposure for the times indicated. **Supplementary Figure S5.** Characterization of the DRE2 mutation in the *AhR *promoter region and expression of the Jdp2 target genes. **Supplementary Figure S6. **EMSA assay of GST–AhR–basic helix-loop-helix (aHLH) binding to DRE2, DRE3 and ARE1 in vitro. **Supplementary Figure S7. **Relative mRNA expression of the AhR target genes *Aldh3a1 *(A), *Cyp1b1 *(B), and *Tiparp *(C) after exposure to DMSO and TCDD in WT and *Jdp2*−/− MEFs. **Supplementary Figure S8.** Uncropped raw data of western blots which were used in this article. **Supplementary Table S1.** Antibodies used in this study. **Supplementary Table S2.** Oligonucleotides. **Supplementary Table S3.** Mutation of ARE1, ARE2, DRE1, DRE2, and DRE3 in AhR promoter. **Supplementary Table S4.** siRNAs used in this study. **Supplementary Table S5.** Experimental models: organisms/strains. **Supplementary Table S6.** Experimental models: cell line. **Supplementary Table S7.** Critical commercial assay. **Supplementary Table S8.** Chemicals, peptides, and recombinant proteins. **Supplementary Table S9.** Recombinant DNA **Supplementary Table S10.** Software and algorithms.

## Data Availability

The datasets generated and analyzed for the current study are available from the corresponding author on reasonable request. The source data are provided as a Source Data file.

## Abbreviations

AhR: Aryl hydrocarbon receptor
AP-1: Activation protein 1
ARE: Antioxidative response element
Arnt: Aryl hydrocarbon receptor nuclear translocator
bHLH: Basic helix-loop-helix
Cyp: Cytochrome P450
DRE: Dioxin responsive element
GSH: Glutathione
GST: Glutathione S-transferase
HRP: Horseradish peroxidase
iPSCs: Induced pluripotent stem cells
Jdp2: Jun dimerization protein 2
Keap1: Kelch-like ECH-associated protein 1
Maf: Musculoaponeurotic fibrosarcoma
MEFs: Mouse embryonic fibroblasts
NAC: N-acetyl-L cysteine
NQO1: NAD(P)H quinoneoxidoreductase1
Nrf2: Nuclear factor (erythroid-derived 2)-like 2
qPCR: Quantitative polymerase chain reaction
ROS: Reactive oxygen species
tBHQ: Tert-butylhydroquinone
TCDD: 2,3,7,8-Tetrachlorodibenzo-*p*-dioxin
UGT: UDP-glucanosyltransferase
WT: Wild type
