# Supplementary material for: Jdp2 is a spatiotemporal transcriptional activator of the AhR via the Nrf2 gene battery
Source: Inflamm Regen. 2023 Aug 18;43:42. doi: 10.1186/s41232-023-00290-6 (PMC10436584; doi:10.1186/s41232-023-00290-6)
Supplement: Supplementary file 1 — Additional file 1: Supplementary Figure S1. Luciferase activity driven by the Cyp1b1 promoter in response to TCDD. Supplementary Figure S2. Characteristics of AhR protein expression and effects of Jdp2 on AhR expression. Supplementary Figure S3. Characterization of AhR promoter activity. Supplementary Figure S4. Interaction of the AhR–Jdp2–Nrf2 axis in nuclei and cytoplasm in WT and Jdp2–/–MEFs in response to TCDD or DMSO exposure for the times indicated. Supplementary Figure S5. Characterization of the DRE2 mutation in the AhR promoter region and expression of the Jdp2 target genes. Supplementary Figure S6. EMSA assay of GST–AhR–basic helix-loop-helix (aHLH) binding to DRE2, DRE3 and ARE1 in vitro. Supplementary Figure S7. Relative mRNA expression of the AhR target genes Aldh3a1 (A), Cyp1b1 (B), and Tiparp (C) after exposure to DMSO and TCDD in WT and Jdp2−/− MEFs. Supplementary Figure S8. Uncropped raw data of western blots which were used in this article. Supplementary Table S1. Antibodies used in this study. Supplementary Table S2. Oligonucleotides. Supplementary Table S3. Mutation of ARE1, ARE2, DRE1, DRE2, and DRE3 in AhR promoter. Supplementary Table S4. siRNAs used in this study. Supplementary Table S5. Experimental models: organisms/strains. Supplementary Table S6. Experimental models: cell line. Supplementary Table S7. Critical commercial assay. Supplementary Table S8. Chemicals, peptides, and recombinant proteins. Supplementary Table S9. Recombinant DNA Supplementary Table S10. Software and algorithms. [file 41232_2023_290_MOESM1_ESM.zip › SInformations, Inflamm and Regener Final 20230601.pdf]

Supplementary Information

Jdp2 is a spatiotemporal transcriptional activator of the AhR via the Nrf2 gene battery

Kenly Wuputra[1-3], Ming-Ho Tsai[1-3], Kohsuke Kato[4], Chia-Chen Ku[1-3], Jia-Bin Pan[1-3], Ya-Han Yang[2,3,5], Shigeo Saito[6], Chun-Chieh Wu[7], Ying-Chu Lin[8], Kuang-Hung Cheng[9], Kung-Kai Kuo[2,3,5], Michiya Noguchi[10], Yukio Nakamura[10], Tohru Yoshioka[1], Deng-Chyang Wu[2,3,11], Chang-Shen Lin[1,9*], and Kazunari K. Yokoyama[1-3 *]

[1] Graduate Institute of Medicine, Kaohsiung Medical University, Kaohsiung 80708, Taiwan.

[2] Regenerative Medicine and Cell Therapy Research Center, Kaohsiung Medical University, Kaohsiung 80708, Taiwan.

[3] Cell Therapy and Research Center, Kaohsiung Medical University Hospital, Kaohsiung 80756, Taiwan.

[4] Department of Infection Biology, Graduate School of Comprehensive Human Sciences, the University of Tsukuba, Tsukuba 305-8577, Japan.

[5] Division of General & Digestive Surgery, Department of Surgery, Kaohsiung Medical University Hospital, Kaohsiung 80756, Taiwan.

[6]Saito Laboratory of Cell technology, Yaita 32901571, Tochigi, Japan.

[7]Department of Pathology, Kaohsiung Medical University Hospital, Kaohsiung 80756, Taiwan.

[8] School of Dentistry, Kaohsiung Medical University, Kaohsiung 80708, Taiwan.

[9] Department of Biological Sciences, National Sun Yat-sen University, Kaohsiung 80424, Taiwan.

[10] Cell Engineering Division, BioResource Research Center, Tsukuba, Ibaraki 305-0074, Japan.

[11] Division of Gastroenterology, Department of Internal Medicine, Kaohsiung Medical University

Hospital 80756, Taiwan.

Tohru Yoshioka is deceased.

*Authors to whom correspondence should be addressed; Kazunari K. Yokoyama and Chang-Shen Lin (Graduate Institute of Medicine, Kaohsiung Medical University, Tel; +886-07312-1101, ext. 2729, FAX +886-7313-3849, e-mail; kazu@kmu.edu.tw, http://orcid.org/0000-0001-8505-7582; changshen.lin@kmu.edu.tw, http://orcid.org/0000-0001-7415-2187).

[Contents]

1. **Methods**
2. **Legends of Supplementary Figures**
3. **Supplementary Figures**
4. **Supplementary Tables**

Materials and methods

shRNA- and siRNA-mediated gene knockdown

Recombinant shRNA lentiviruses against mouse *AhR, Nrf2, MafK, Arnt, GFP, and Jdp2* were obtained from the siRNA Core Center at Academia Sinica (Taipei, Taiwan). The predesigned ON-TARGETplus SMARTpool siRNA against mouse *AhR, ARNT, Ahrr, MafK,* and *Nrf2* and a control scrambled siRNA were purchased from GE Dharmacon (Austin, TX, USA). MEFs were seeded into a six-well (for Western blotting) or 24-well plate (for the luciferase reporter assay) and transfected in the presence of 20–40 nM of either siRNA or negative-control RNA in a final volume of 0.5 mL (24-well plate) or 2 mL (six-well plate) of OPTI-MEM with Lipofectamine RNAiMAX (both from

Invitrogen). shRNA was transfected into MEFs at a multiplicity of infection of 10. After 24 h, fresh culture medium containing 10% FBS was added, the cells were transfected with the luciferase plasmids, and the luciferase reporter assay was performed as described above. To confirm the knockdown efficiency of shRNA and siRNA, cells were harvested at 48 and 72 h after shRNA infection and siRNA transfection, respectively, and analyzed by immunoblotting and other methods.

Isolation of RNA and qPCR

Total RNA was purified using a PureLink™ RNA Mini Kit (Invitrogen). RNA was reverse transcribed to cDNA using SuperScript III Reverse Transcriptase (Invitrogen). Real-time PCR was performed on a StepOne or ABI7500 PCR instrument (Applied Biosystems, Foster City, CA, USA) using Applied Biosystems Fast SYBR® Green Master mix in 20 μL reaction volumes. The threshold cycle *Ct* values were averaged from technical duplicates. The transcript level of each gene was normalized to that of *GAPDH*. The $2^{-\Delta\Delta Ct}$ method was used to calculate relative gene expression level, and expression was normalized to the mRNA level in DMSO-treated WT MEFs, which was taken as 1.0. Data are expressed as mean ± SEM of three biological replicates. The forward and reverse primers used were listed in **Supplementary Table 2**.

Electrophoresis mobility shift assay

Five micrograms of GST fusion protein (GST-AhR-bHLH from H. Ito) were incubated at 25°C for 30 min with T4 kinase-labeled mouse *AhR*-DRE1, 2, and 3, and ARE1 oligonucleotides (see **Supplementary Table 2**). The products were resolved at 4 °C on a 5% nondenaturing polyacrylamide gel in 0.5× Tris-borate/EDTA buffer, exposed to a radioactive imaging plate, and detected on an FLA-2000 machine (Fuji Photo Film, Tokyo, Japan) as described previously [1].

Coimmunoprecipitation and Western blot analysis

Whole-cell lysates were prepared using PRO-PREP™ protein extraction solution (iNtRON

Biotechnology). Subcellular fractionation was performed using an NE-PER® Nuclear and Cytoplasmic Extraction kit (Thermo Scientific). Protein concentration was measured using the Bradford method with bovine serum albumin as the standard (Bio-Rad). For coimmunoprecipitation, 300 μg of extract was precleared with Protein G Agarose beads (Millipore) at 4°C for 1 h and then incubated with 1 μg of IP-antibody or preimmune IgG at 4°C overnight with rotation. The immune complexes were precipitated with protein G beads and analyzed by Western blotting, as described below. The immunoprecipitated proteins were dissolved in 30 μL of 2× Laemmli buffer, boiled for 5 min, and analyzed by Western blotting.

To measure MLC phosphorylation, MEFs were deprived of serum overnight, treated with DMSO with or without TCDD for 6 h, and lysed with 10% trichloroacetic acid. Precipitated protein was collected by centrifugation, washed in absolute ethanol three times each for 5 min, and solubilized completely in urea buffer (8 M urea, 20 mM Tris, 23 mM glycine, and 0.2 mM EDTA). Equal amounts of protein were electrophoresed on an acrylamide gel and then transferred to nitrocellulose. After blocking with 5% nonfat dry milk in Tris-buffered saline containing 0.1% Tween 20 for 1 h at room temperature, the membranes were blotted with the primary antibody at 4°C overnight with shaking and then incubated with the horseradish peroxidase-conjugated secondary antibody for 1 h at room temperature. Immunoreactive bands were visualized with Immobilon Western Chemiluminescent HRP Substrate (Millipore), and images were captured using a ChemiDoc-XRS^{+} apparatus (Bio-Rad Laboratories) and quantified using Image Lab software (version 4.1). The primary antibodies used were to the following proteins: AhR (1:1000, sc-8088), Nrf2 (1:1000, sc-722), Arnt (1:1000, CST5537), p-MLC2^{Ser19} (1:1000, CST3671), MLC2 (1:1000, CST3672), JDP2 (1:1000, from Dr. Aronheim or sc-367695), MafK (1:1000, sc-477), lamin A/C (1:1000, sc-6215), and β-actin (1:5000, sc-81178).

Statistical analyses

Data are shown as the mean ± SEM. The results were compared between experimental conditions

using GraphPad Prism 5.0 software (GraphPad Software, San Diego, CA, USA). For multiple comparisons, one-way analysis of variance (ANOVA) followed by the Tukey *post hoc* test or two-way ANOVA with the Bonferroni *post hoc* test was used. Student's unpaired two-tailed *t* test was used to compare the control and treatment groups. Student's paired one-tailed *t* test was used to identify each site-directed mutagenesis site in the *AhR* promoter. Mann–Whitney nonparametric median statistical analysis was used to compare cell areas. All differences were considered to be significant at $p \leq 0.05$.

References

1. Tanigawa S, Lee CH, Lin CS, Ku CC, Hasegawa H, Qin S, Kawahara A, Korenori Y, Miyamori K, Noguchi M *et al*. Jun dimerization protein 2 is a critical component of the Nrf2/MafK complex regulating the response to ROS homeostasis. Cell Death & Disease 2013; 4(11):e921-e921.

Supplementary Figures

Supplementary Fig. 1

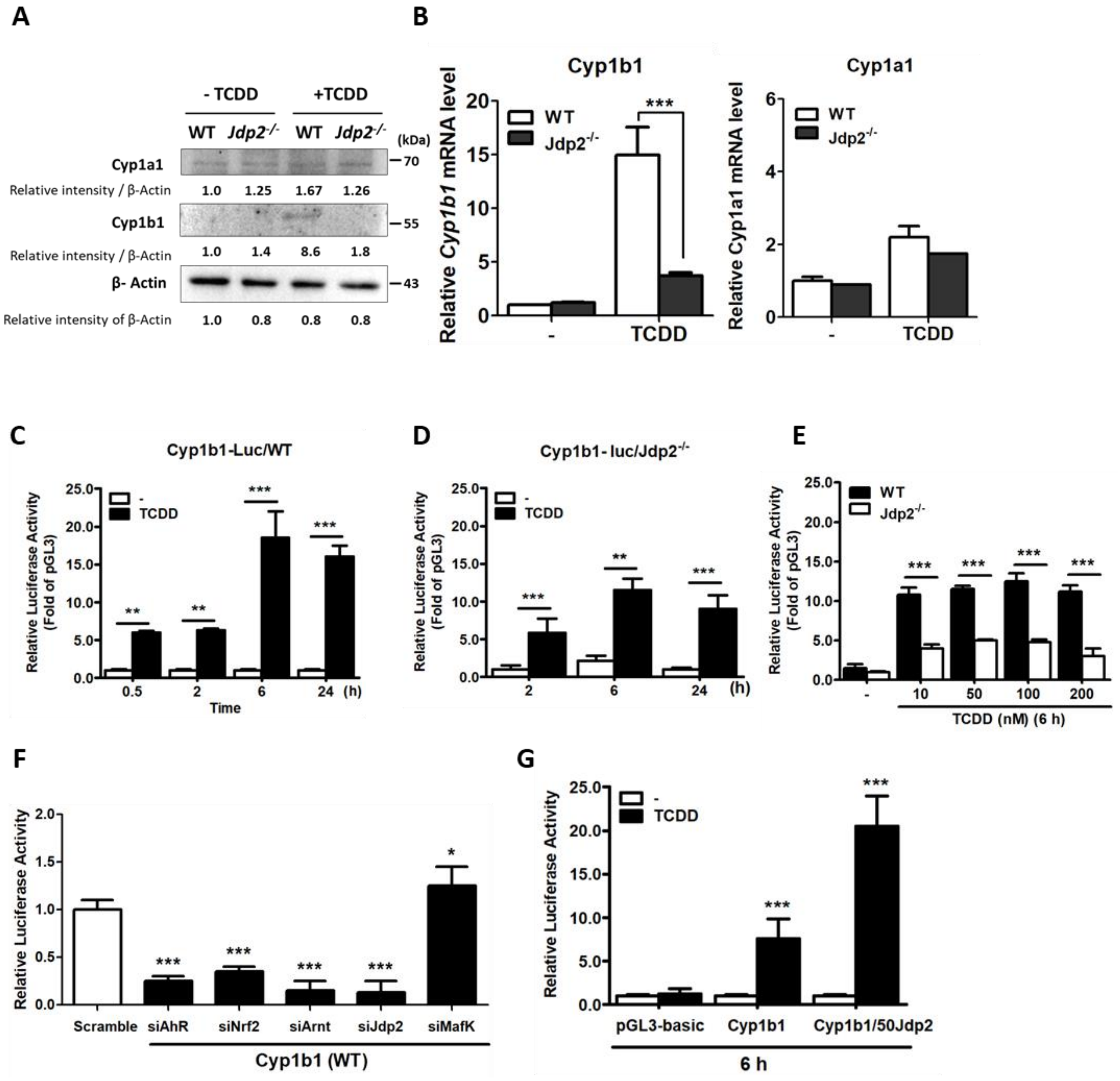

Supplementary Fig. 1. Luciferase activity driven by the *Cyp1b1* promoter in response to TCDD. **(A)** Expression of Cyp1a1 and Cyp1b1 proteins in WT and *Jdp2*$^{-/-}$ MEFs in the presence or absence of TCDD. The relative expression ratio was calculated based on β-actin expression. Cropped figures are shown. See **Supplementary Fig. 8** for the original full-length blot images. The intensity of each band was then quantified. The relative value was normalized to β-Actin and shown as ratio. **(B)** Expression of *Cyp1a1* and *Cyp1b1* mRNAs in WT and *Jdp2*$^{-/-}$ MEFs. The promoter activity is expressed as a relative value (fold) to the pGL3 basic vector (***p < 0.001). (**C, D)** Effects of time of TCDD exposure on promoter activity in WT (C) and *Jdp2*$^{-/-}$ MEF (D) cells. *pGL3-CYP1B1* luciferase (0.5 μg) and *pRL-CMV-Renilla* (0.01 μg) plasmids were cotransfected into MEFs. **(E)** Luciferase plasmid-transfected MEFs were treated with different doses of TCDD or 0.1% DMSO for 6 h. **(F)** Effects of siRNA against *AhR*, *Nrf2*, *Arnt*, *Jdp2*, and *MafK*, and the scrambled control on *Cyp1b* promoter luciferase activity were examined. The value for the scrambled control was set at 1.0. **(G)** Jdp2 increased the *Cyp1b1* promoter luciferase activity in the presence of 10 nM TCDD. The promoter activity is expressed as relative value (fold) to the *pGL3* basic vector. The above data are mean ± SEM from four independent measurements, analyzed using two-way ANOVA with the Bonferroni *post hoc* test for multiple comparisons (*p < 0.05, **p < 0.01, ***p < 0.001).

Supplementary Fig. 2

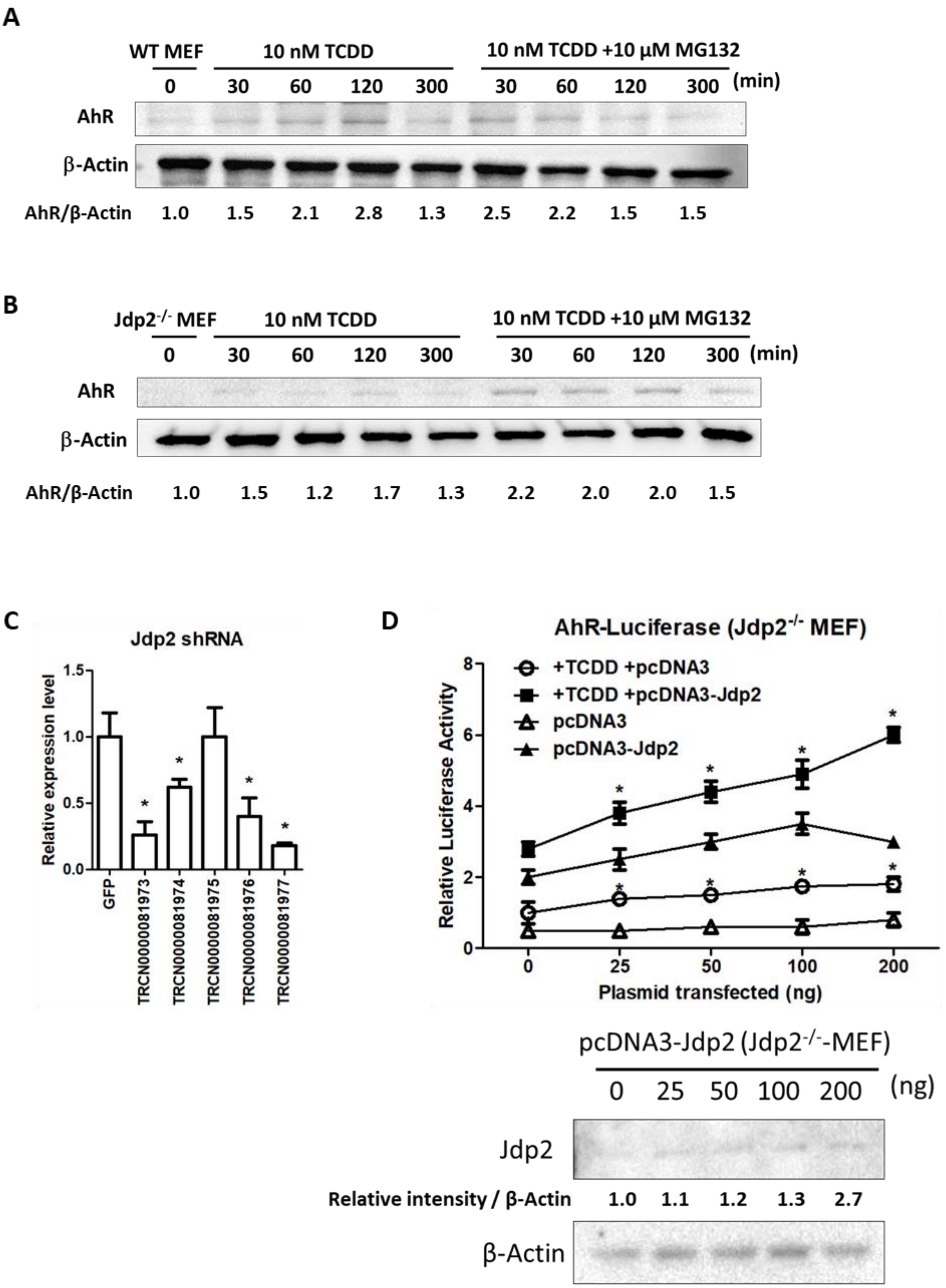

Supplementary Fig. 2. Characteristics of AhR protein expression and effects of Jdp2 on AhR expression. (**A, B)** Effects of MG132 on AhR expression in response to TCDD in WT (A) and *Jdp2*$^{-/-}$ MEFs (B). The time course of the response to 10 nM of TCDD with or without 10 μM MG132 was measured in WT and *Jdp2*$^{-/-}$ MEFs exposed for indicated time. Expression of the AhR relative to that of β-actin was calculated. **(C)** Effects of various constructs of shJdp2 on *Jdp2* mRNA expression were examined in WT MEFs. **(D)** Effects of increased dose of *pcDNA3-Jdp2* on *AhR* promoter luciferase activity with or without TCDD (10 nM) in *Jdp2*$^{-/-}$ MEFs. Expression of Jdp2 was examined by Western blotting with anti-Jdp2 antibodies. Western blotting cropped figures are shown. See **Supplementary Fig. 8** for the original full-length blot images. The intensity of each band was then quantified. The relative value was normalized to β-Actin and shown as ratio. All above data represent the mean ± SEM (n = 5) (*$p < 0.05$) as indicated by two-way ANOVA with the Bonferroni *post hoc* test for multiple comparisons.

Supplementary Fig. 3

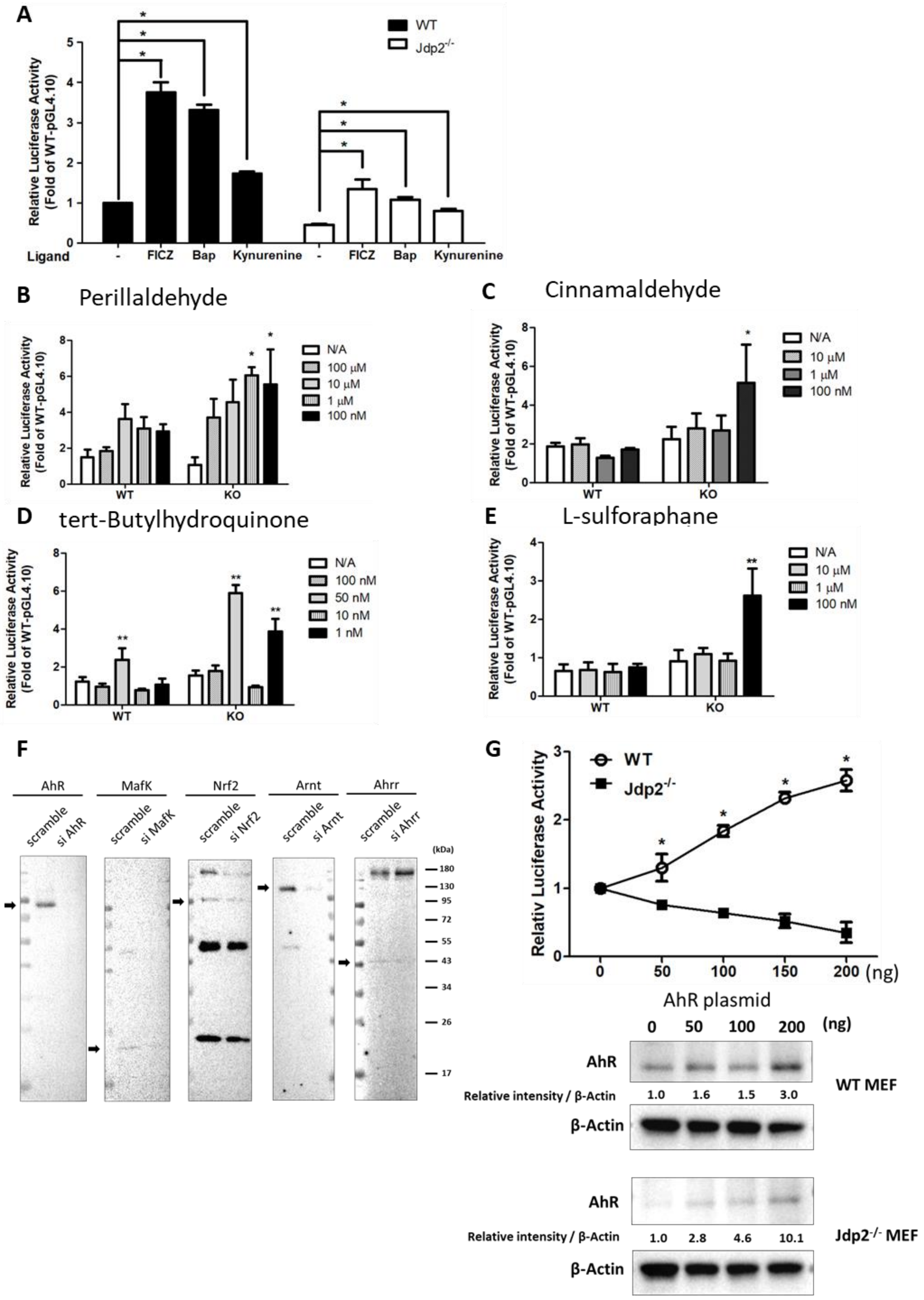

Supplementary Fig. 3. Characterization of *AhR* promoter activity.

(A) Effects of phase I reagents on *AhR* promoter luciferase activity in WT and *Jdp2*$^{-/-}$ MEFs. 6-Formylindolo[3,2-*b*]carbazole (FICZ), benzo[*a*]pyrene (BaP), and kynurenine were added to WT and *Jdp2*$^{-/-}$ MEFs at a concentration of 100 nM for each, and the cells were incubated for 2 h and harvested for the luciferase assay of the *AhR* promoter (*p < 0.05). (**B-E)** Effects of phase II reagents in WT and *Jdp2*$^{-/-}$ MEFs. Cells were exposed for 2 h to perillaldehyde (B), cinnamaldehyde (C), tert-butylhydroquinone (D), or L-sulforaphane (E) at the indicated doses, and the *AhR* promoter luciferase activity was measured and expressed relative to the pGAL4.10 luciferase control (*p < 0.05, **p < 0.01). **(F)** Effects of siRNA against *AhR, MafK, Nrf2, Arnt,* and *Ahrr* in WT-MEFs. Western blot analysis was performed to evaluate the expression of AhR- and Nrf2-related proteins. The control shows the effects of Sc-44234 (Santa Cruz Biotechnology) on the expression of each protein (see STable 4). A representative example is shown. **(G)** Effects of increased doses of AhR on *AhR* promoter luciferase activity in WT and *Jdp2*$^{-/-}$ MEFs. The AhR dose was 50, 100, 50, or 200 ng, and the *AhR* promoter luciferase activity was measured as described in the Materials and methods. Expression levels of AhR proteins were examined by Western blotting in WT and *Jdp2*$^{-/-}$ MEFs (*p< 0.05). Western blotting cropped figures are shown. See **Supplementary Fig. 8** for the original full-length blot images. The intensity of each band was then quantified. The relative value was normalized to β-Actin and shown as ratio. All above data represent the mean ± SEM (*n* = 5). Statistic analyzed by two-way ANOVA with the Bonferroni *post hoc* test for multiple comparisons.

Supplementary Fig. 4

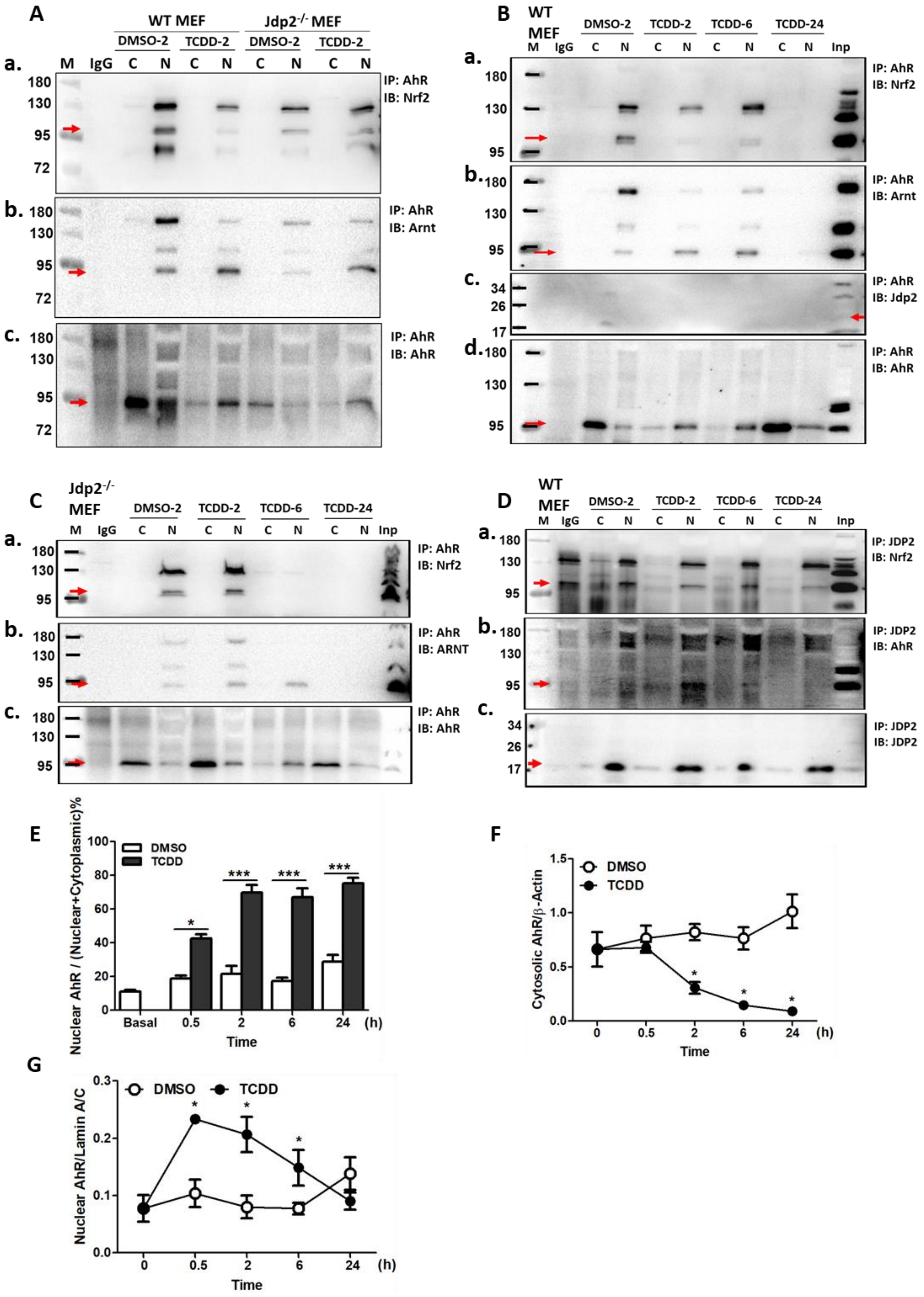

Supplementary Fig. 4. Interaction of the AhR–Jdp2–Nrf2 axis in nuclei and cytoplasm in WT and *Jdp2*$^{-/-}$ MEFs in response to TCDD or DMSO exposure for the times indicated. **A** Cell lysates from cytosolic and nuclear fractions of WT and *Jdp2*$^{-/-}$ MEFs after exposure to TCDD for 2 h were immunoprecipitated with antibodies against AhR **a.** Immunoprecipitation (IP): AhR, immunoblot (IB): Nrf2, **b.** IP: AhR, IB: Arnt **c.** IP: AhR, IB:AhR. **B** Cell lysates from cytosolic and nuclear fractions from WT MEFs after exposure to TCDD for 2, 6, or 24 h were immunoprecipitated with antibody against AhR **a.** IP: AhR, IB: Nrf2, **b.** IP: AhR, IB: Arnt, **c.** IP: AhR, IB: Jdp2, **d.** IP: AhR, IB: AhR. **C** Cell lysates from cytosolic and nuclear fractions from *Jdp2*$^{-/-}$ MEFs after exposure to TCDD for 2, 6, or 24 h were immunoprecipitated with antibody against AhR **a.** IP: AhR, IB: Nrf2, **b.** IP: AhR, IB: Arnt **c.** IP: AhR, IB: AhR. **D** Cell lysates from cytosolic and nuclear fractions from WT MEFs after exposure to TCDD for 2, 6, or 24 h were immunoprecipitated with antibody against Jdp2, and the bound proteins were blotted with Nrf2, AhR, and Jdp2 antibodies **a.** IP: AhR, IB: Nrf2, **b.** IP: AhR, IB: Arnt **c.** IP: AhR, IB: AhR. IgG was used as a negative control. The red arrows indicate the targeted proteins in each panel. The nontargeted protein bands should be examined further to determine whether they are real complexed components or nonspecific binding proteins. **E** Relative localization ratio of AhR protein in the nucleus and cytoplasm in WT MEFs after exposure to DMSO and TCDD for 30 min, 2 h, 6 h, or 24 h (*$p < 0.05$; ***$p < 0.005$). **F**, **G** Relative localization ratio of AhR protein in WT MEFs after exposure to TCDD and DMSO for 30 min, 2 h, 6 h, or 24 h. The ratio was generated based on the β-actin expression **(F)** or lamin C expression **(G)** levels (*$p < 0.05$). All values are expressed as the mean ± SEM ($n = 5$). The data were analyzed using two-way ANOVA with the Bonferroni post hoc test for multiple comparisons.

Supplementary Fig. 5

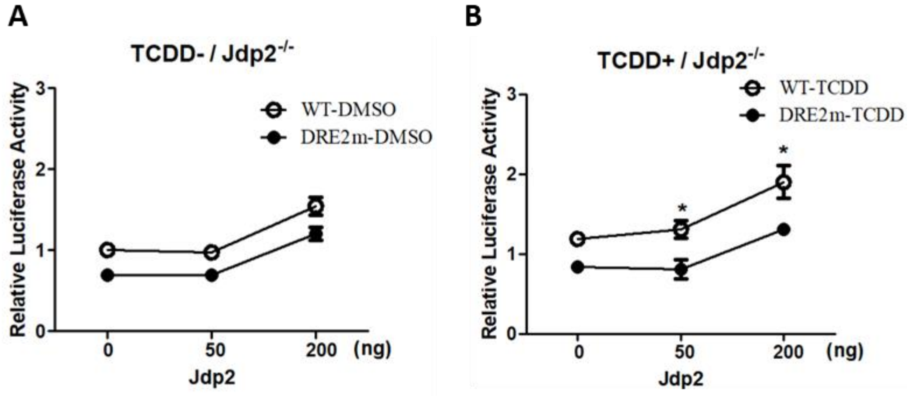

Supplementary Fig. 5. Characterization of the DRE2 mutation in the *AhR* promoter region and expression of the Jdp2 target genes. Effects of DRE2 mutation in the *AhR* promoter in the absence **(A)** or presence **(B)** of TCDD exposure in *Jdp2*$^{-/-}$ MEFs with increased Jdp2 doses of 50 and 200 ng (*p < 0.05).

Supplementary Fig. 6

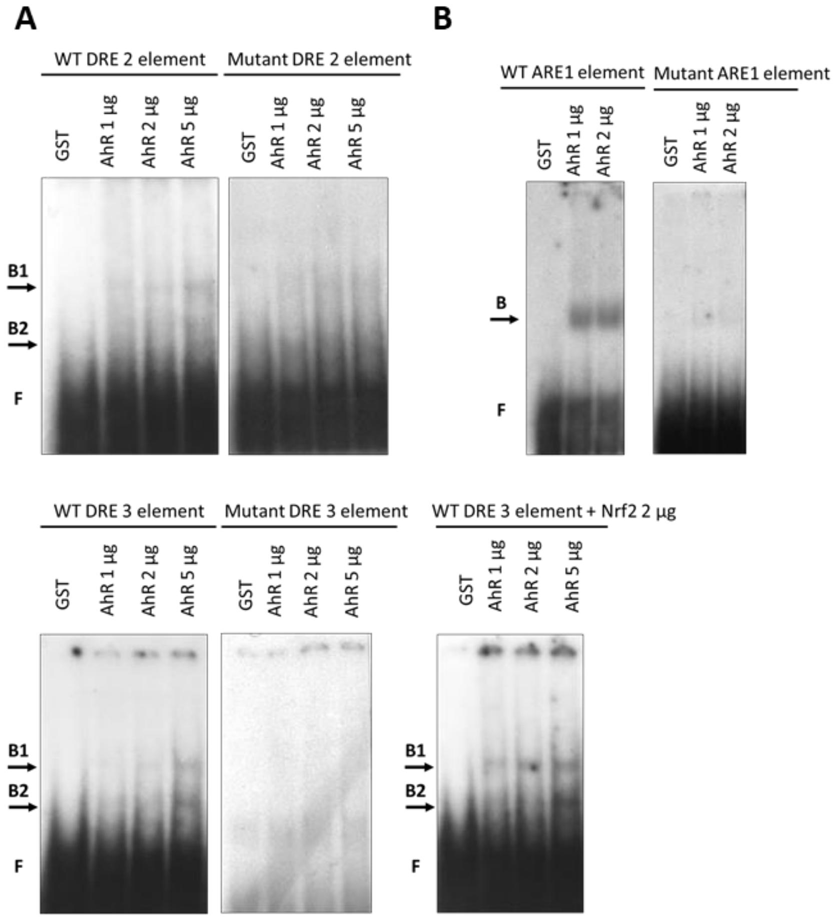

Supplementary Fig. 6. EMSA assay of GST–AhR–basic helix-loop-helix (bHLH) binding to DRE2, DRE3 and ARE1 *in vitro*. **(A)** DNA binding of GST–AhR–bHLH protein (Hideaki Ito, Akita University, Japan) to DNA probe DRE2 and its mutant DRE2. **(B)** DNA binding of GST–AhR protein to the ARE1 and its mutant probe. (**C**) DNA binding of GST–AhR protein to the DRE3 and its mutant probe. The nucleotide sequences of these probes are listed in Supplementary Table 3. EMSA reactions were performed for the respective DNA probes as described in the Methods section using [γ-^{32}P]-labeled double-stranded DRE2 or DRE3 or ARE1 oligonucleotides, GST–AhR–bHLH were purified using GST affinity resins. "B" indicates the DNA-protein complexes and "F" indicates the respective DNA-free probes.

Supplementary Fig. 7

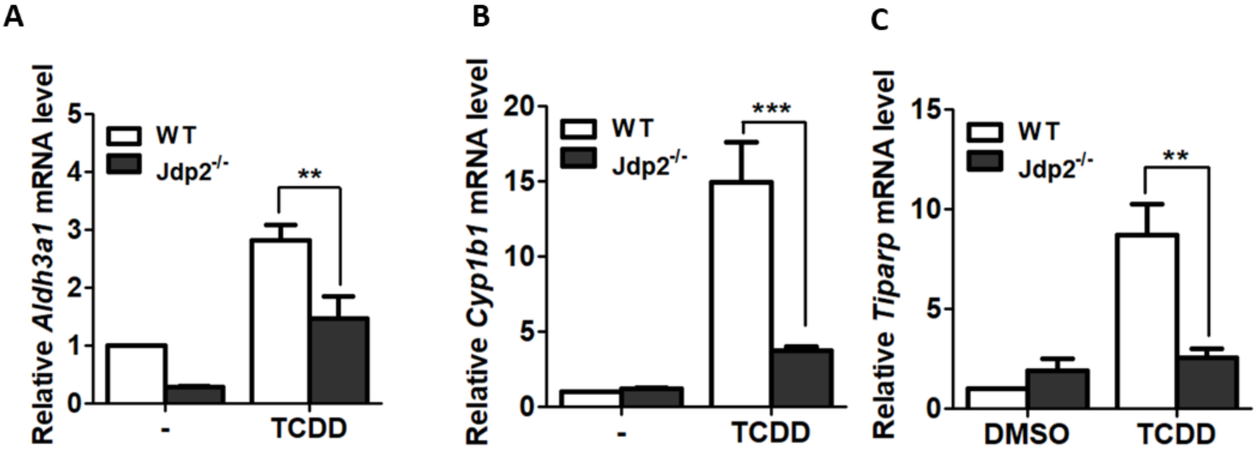

Supplementary Fig. 7. Relative mRNA expression of the AhR target genes *Aldh3a1* (A), *Cyp1b1* (B), and *Tiparp* (C) after exposure to DMSO and TCDD in WT and *Jdp2*$^{-/-}$ MEFs. Data were obtained using qPCR analysis, as described in the Materials and methods. The mRNA expression of each transcript in WT MEFs in response to DMSO was set at 1.0 (**$p < 0.01$, ***$p < 0.005$). All above data represent the mean ± SEM ($n = 5$). Statistic analyzed as indicated by two-way ANOVA with the Bonferroni *post hoc* test for multiple comparisons.

Supplementary Fig.8

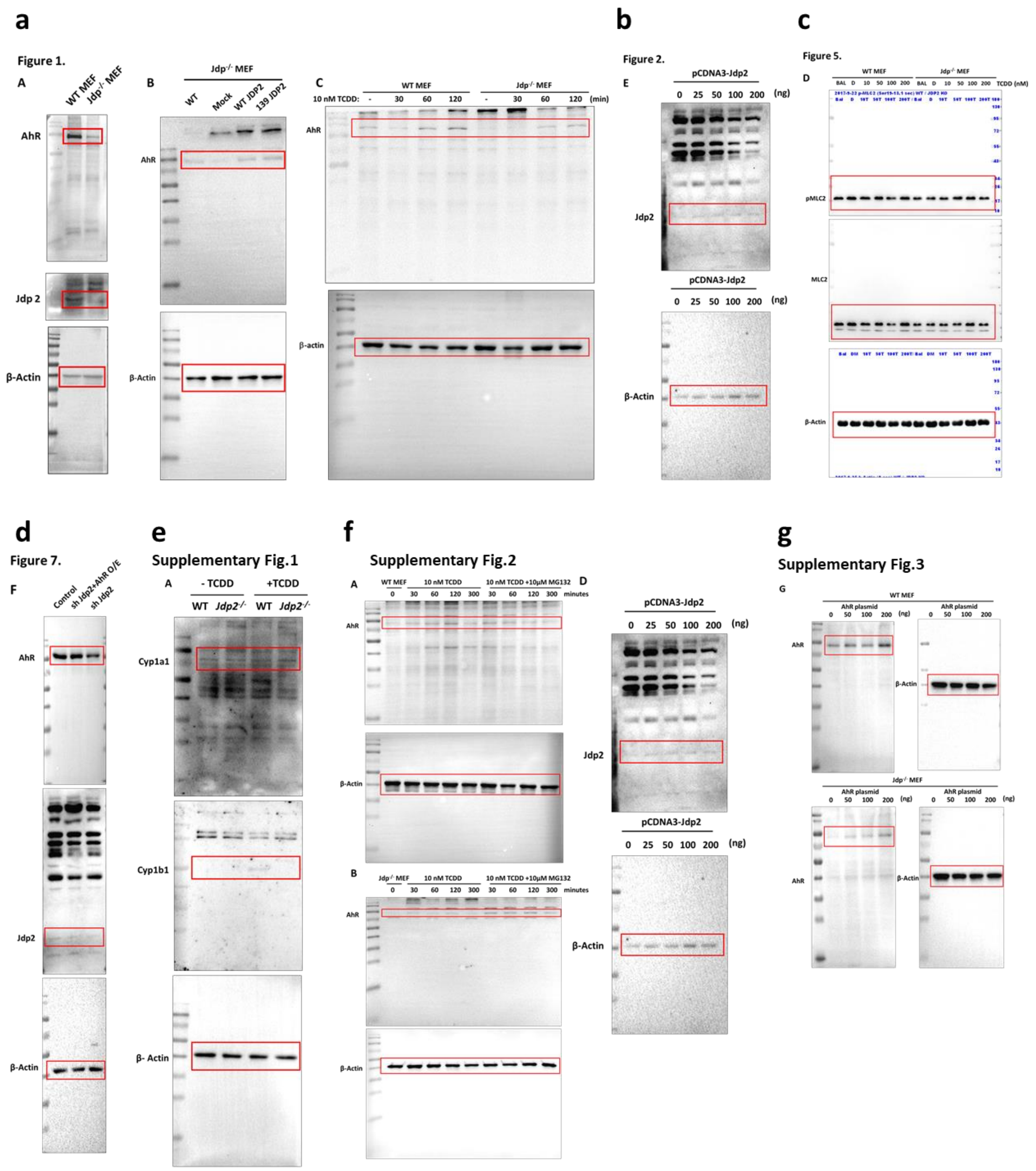

Supplementary Fig. 8. Uncropped raw data of western blots which were used in this article.

(a-g) Results were provided according to the sequence mentioned in main text, **(a)** Figure 1A-C, **(b)** Figure 2E, **(c)** Figure 5D, **(d)** Figure 7F, **(e)** Supplementary Figure 1A, **(f)** Supplementary Figure 2A, B, and D, **(g)** Supplementary Figure 3G. The red rectangles represented regions which were cropped to use in the figures of this text and supplementary data.

Supplementary Tables

S Table1. Antibodies used in this study

Antibody name	Company	Cat. No.	RRIDs
Arnt	Cell Signaling Technology	CST#5537	AB_10694232
HIF-1beta	Gene Tex	GTX128795	AB_2861418
AhR	Santa Cruz Biotechnology	SC-8088	AB_2223957
AhR	Santa Cruz Biotechnology	SC-133088	AB_2273721
Nrf2	Santa Cruz Biotechnology	SC-722	AB_2108502
Nrf2	Gene Tex	GTX103322	AB_1950993
Nrf2	Cell Signaling Technology	CST#14596	AB_2798531
Mafk (NF-E2p18)	Santa Cruz Biotechnology	SC-477	AB_2137821
Jdp2	A gift from Dr. A. Aronheim		
Jdp2	Santa Cruz Biotechnology	SC-517133	AB_2861419
Ahrr	Sigma-Aldrich	HpA019614	AB_1855109
β-actin	Santa Cruz Biotechnology	SC-47778	AB_2714189
FLAG-M2	Merck Millipore	F1804	AB_262044
MLC2	Cell Signaling Technology	CST#3672	AB_10692513
pMLC2	Cell Signaling Technology	CST#3671	AB_330248
GAPDH	Millipore	MAB374	AB_2107445
Phalloidin (=F-actin) Alexa-Fluor488-Phalloidin	Thermo Fisher Scientific	A12379	N/A
Normal Rabbit IgG	Cell Signaling Technology	CST#2729	AB_1031062
Normal Mouse IgG	Merck Millipore	12-371	AB_145840
Anti-Rabbit IgG HRP	Cell Signaling Technology	CST#7074	AB_2099233
Anti-Mouse IgG HRP	Cell Signaling	CST#7076	AB_330924

	Technology		
Anti-Goat IgG HRP	Santa Cruz Biotechnology	SC-2020	AB_631728
Alexa-Fluor ® 488 conjugated Goat anti-Mouse IgG	Thermo Fisher Scientific	A-11029	AB_138404
Alexa-Fluor® 488 conjugated Goat anti-Rabbit IgG	Thermo Fisher Scientific	A-11034	AB_2576217
Alexa-Fluor® 488 conjugated Rabbit anti-Goat IgG	Thermo Fisher Scientific	A-11078	AB_2534122
Alexa-Fluor ® 594 conjugated Goat anti-Mouse IgG	Thermo Fisher Scientific	A-11032	AB_2534091
Alexa-Fluor ® 594 conjugated Goat anti-Rabbit IgG	Thermo Fisher Scientific	A-11037	AB_2534095
Alexa Fluor® 647-conjugated Goat anti-rat IgG (H+L)	Cell Signal Technology	#4418	AB_1904017
Annexin V	BD Bioscience	51-65874X	AB_2888981

S Table2. Oligonucleotides

Regions	Primer sequences	Amplified size (bp)
ARE1	Sense 5'-CCTGGTAAATCTTGATGTCTGGG-3' Antisense 5'-ATGACGCAGGACGTAGTGAC-3'	159
ARE2	Sense 5'-CAGAATTTCCACCTTTTCCCACA-3' Antisense 5'-AGGAAAGAACACAGGAGTGC-3'	223
DRE1	Sensei 5'-ACTGCGCGGGGTC	139

	G-3’<br>Antisense 5’-<br>GTCCACCAGTTCG<br>TCCTCC-3’	
DRE2/3	Sense 5’-<br>GACGAACTGGTGG<br>ACGGA-3’<br>Antisense 5’-<br>GGAGAAACCCGC<br>ACGCTA-3’	145
Ahrr	Sense 5′-<br>TAGGAAGAGAAG<br>GAAGCCCATTCA-<br>3′<br>Antisense 5′-<br>GGTGCCGTTTGGA<br>AGGATTTG-3′	
Aldh3a	Sense 5′-<br>TGCTGGAGAGGAC<br>TGTGTAGA-3′<br>Antisense 5′-<br>GGTCGAGTCTTGC<br>CTGAGTT-3′	
Cyp1b1	Sense 5′-<br>TTACGGACATCTT<br>CGGAGCC-3′<br>Antisense 5′-<br>CCCACAACCTGGT<br>CCAACTC-3′	
Tiparp	Sense 5′-<br>ACGAAGGCTGTCT<br>ACACCAC-3′<br>Antisense 5′-<br>CCCGAGAGTTGGC<br>TTCTT CA-3′	
EMSA WT<br>AhR-DRE1	Sense 5′-<br>ACCGGGCGCGGCT<br>AGCGTGCGGGTTT<br>-3′	

	Antisense 5′-GAGAAACCCGCACGCTAGCCGCGCCC -3′	
EMSA mutant AhR-DRE1	Sense 5′-ACCGGGCGCGGCGCTACATCGGGTTT -3′ Antisense 5′-GAGAAACCCGATGTAGCGCCGCGCCC -3′	
EMSA WT AhR-DRE2	Sense 5′-TCGGTGCCCCACGCGTGTCCCGGAGAG -3′ Antisense 5′-AGCCTCTCCGGGACACGCGTGGGGCAC -3′	
EMSA mutant AhR-DRE2	Sense 5′-TCGGTGCCCCACTTCCACGGCGGAGAG -3′ Antisense 5′-AGCCTCTCCGCCGTGGAAGTGGGGCAC -3′	
EMSA WT AhR-DRE3	Sense 5′-CGGGCGGCAGCGTGTGTGTGCGCTCCCTT -3′ Antisense 5′-TCAAAGGGAGCGCACACACACGCTGCCGC -3′	
EMSA mutant AhR-DRE3	Sense 5′-CGGGCGGCACGTACACTGCCACCTCC	

	CTT -3′<br>Antisense 5′-<br>TCAAAGGGAGGT<br>GGCAGTGTACGTG<br>CCGC -3′	
EMSA WT<br>AhR-ARE1	Sense 5′-<br>TTTCCCACAGTGA<br>CTTTCCCAAGAAA<br>GATG -3′<br>Antisense 5′-<br>TTCCATCTTTCTTG<br>GGAAAGTCACTGT<br>GGG -3′	
EMSA mutant<br>AhR-ARE1	Sense 5′-<br>TTTCCCACAACAG<br>TCCCTTCAAGAAA<br>GATG -3′<br>Antisense 5′-<br>TTCCATCTTTCTTG<br>AAGGGACTGTTGT<br>GGG -3′	

S Table3. Mutation of ARE1, ARE2, DRE1, DRE2, and DRE3 in AhR promoter

**Site**	**Primer sequences for mutation cis-element**
ARE1	5’-<br>GGGAGTCACTACGTCCTCTTC<br>CGCACCGTGCTGCGAAGAGG<br>GTG-3’
ARE2	5’-<br>TTCCACCTTTTCCCACAACAG<br>TCCCTTCAAGAAAGATGGAAC<br>ATC-3’
DRE1	5’-<br>GGACCGGGCGCGGCGCTACAT<br>CGGGTTTCTCCTC-3’
DRE2	5’-<br>CTCGGTGCCCCACTTCCACGG<br>CGGAGAGGCTCAGC-3’

DRE3	5'-CGCGGCGGGCGGCACGTACACTGCCACCTCCCTTTGACGCTC-3'
AP-1	5'-CTTCCATCTGTTTTGTTCCCGTACACCAGAATTTCCACCTTTTC-3'

S Table4. siRNAs used in this study

siRNA	Santa Cruz Co.	Other company
Control siRNA	SC-44234	N/A
Negative control	#01 siRNA	Ambion ®. Thermo Fisher Scientific
siAhR	SC-29658	N/A
siAhRR	SC-140918	N/A
siNrf2	SC-37049	N/A

S Table5. Experimental models: organism/ strains

Organism/ strain	Origin
C57/BL6J $Jdp2^{-/-}$	RIKEN BioResource Research Center
C57/BL6J	RIKEN BioResource Research Center

S Table6. Experimental models: cell line

Cell line	Catalog number
C57/BL6J WT mouse embryonic fibroblasts (MEFs)	N/A
C57/BL6J $Jdp2^{-/-}$ mouse embryonic fibroblasts (MEFs)	N/A
HepG2	HB8065™
MCF7	HTB-22™
HEK293T	CRL-3216; PRID-CVCL.0063
HeLa S3	CCL-2.2™
NIH3T3	CRL-1658

S Table7. Critical commercial assay

Commercial kit	Company	Catalog number
KAPA HiFi PCR kit	KAPABIOSYSTEMS	KR0368-v13.19
Quick-Change Lighting Site-Directed Mutagenesis kit	Agilent Technologies	210513

PRO-PREPTM protein extraction solution	iNtRON Biotechnology	17081
NE-PER®Nuclear and cytoplasmic extraction kit	Thermo scientific	78833
GSH assay kit	Cayman Chemical Co.,	703002
ROS-GloTM H2O2 assay	Promega Co.	G8820

S Table8. Chemicals, peptides, and recombinant proteins

Commercial product	Company	Catalog number
Dimethyl sulfoxide (DMSO)	Sigma-Aldrich	D8418
Benzo[α]pyrene	Sigma-Aldrich	B1760
Tert-benzo hydroquinone (tBHQ)	Sigma-Aldrich	DHR3512
L-sulforaphane (L-SNF)	Sigma-Aldrich	S6317
6-firmyllindole [3,2-b]carbazole (FICZ)	Sigma-Aldrich	SML1489
L-Kynurenine	Sigma-Aldrich	K8625
2,3,7,8-terrachlorodibenzo-*p*-dioxin (TCDD)	AccuStandard	D-404N
4'-,6-diamino-2-phenylindole (DAPI)	Sigma-Aldrich	D9542
ProLong®Gold antifade mountant	Thermo Fisher scientific	P10144
Polyvinylidene difluoride membrane (PVDF)	Thermo Fisher scientific	88518
Lipofectamine 2000	Invitrogen	11668-50
Polyethylenimine Linear MW 25,000	Polysciences Inc.	23966
Protein A/G agarose beads	Merck Millipore	16-266
Proteinase K	Promega	EO0491
CM-H2DCFDA	Life Technologies	C-6827
Matrigel Matrix	Corning	356234
Mitomycin C	FUJFILM	JAN 4548995058638

S Table9. Recombinant DNA

Recombinant DNA	Origin
pcDNA-Nrf2	Tanigawa et al. 2013
pcDNA-Mafk	Tanigawa et al. 2013

pcDNA-Jdp2	Tanigawa et al. 2013
pcDNA-Jdp2A139C	This paper
pCMV S-FLAG	Pan et al. 2010
pQCIN-CA-AhR-EGFP	This paper
pCYP1b-luciferase	This paper
pPyCAG-BstXI-IRES-Zeocin-pA	This paper
pPyZmJdp2	This paper
pCAG-HIVgp	Tanigawa et al. 2013
pCMV-VSV-G-RSV-Rev	Tanigawa et al. 2013
CSII-CMV-MCS-IRES2-Bsd	Tanigawa et al. 2013
pGL4.1	Promega
pRL-CMV-Renilla luciferase	Tanigawa et al. 2013
pRL-CMV-Firefly luciferase	Tanigawa et al. 2013

S Table10. Software and algorithms

Software and algorithms	Website
ALGGEN-PROMO	http://alggen.lsi.upc.es/
Image Lab software version 4.1	https://www.bio-rad.com/en-tw/product/image-lab-software
Image J	https://imagej.nih.gov/ij/
