# Supplementary figures and images for: Jdp2 is a spatiotemporal transcriptional activator of the AhR via the Nrf2 gene battery
Source: Inflamm Regen. 2023 Aug 18;43:42. doi: 10.1186/s41232-023-00290-6 (PMC10436584; doi:10.1186/s41232-023-00290-6)

**Figure 1.**

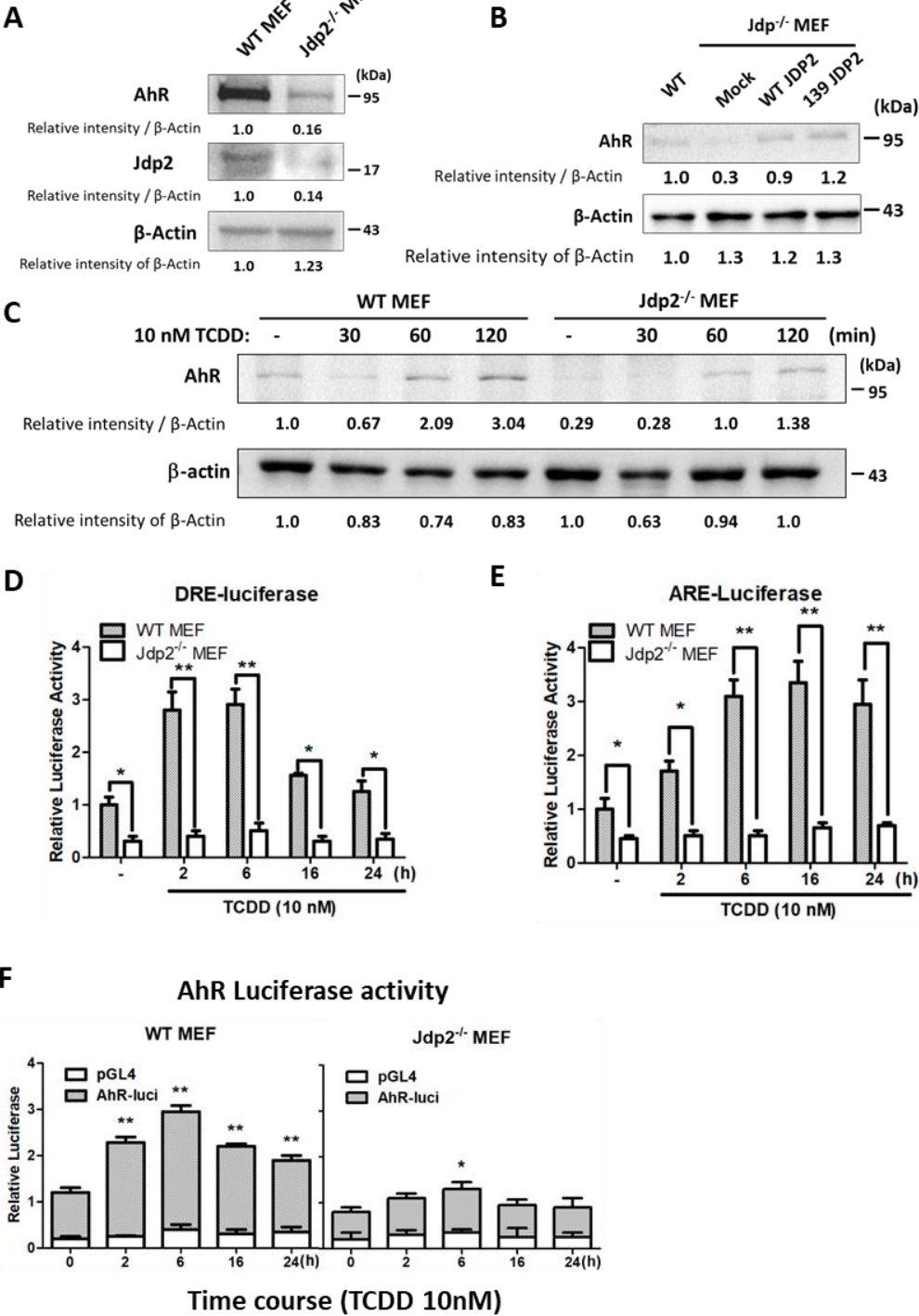

**Figure 2.**

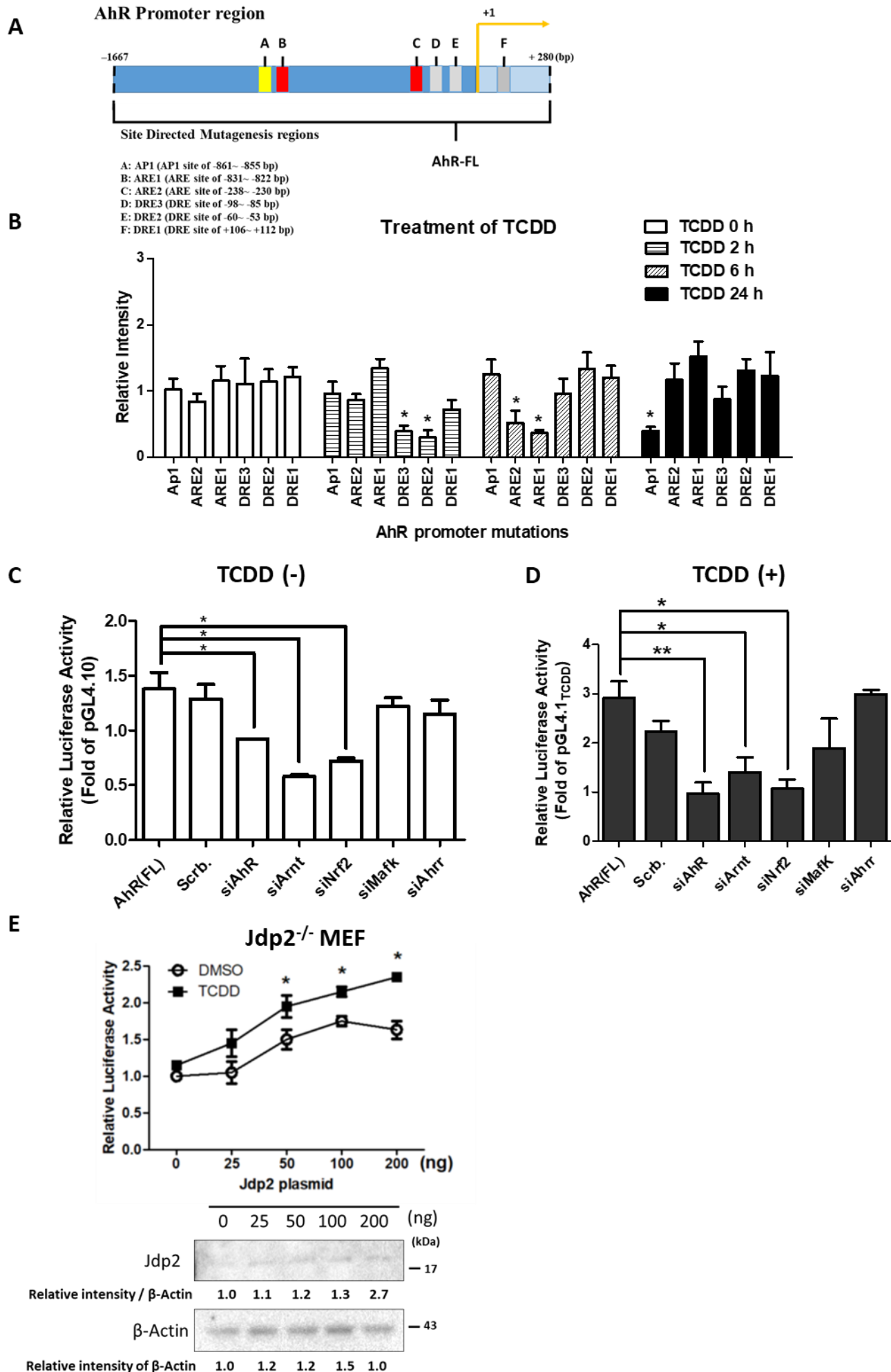

Figure 3.

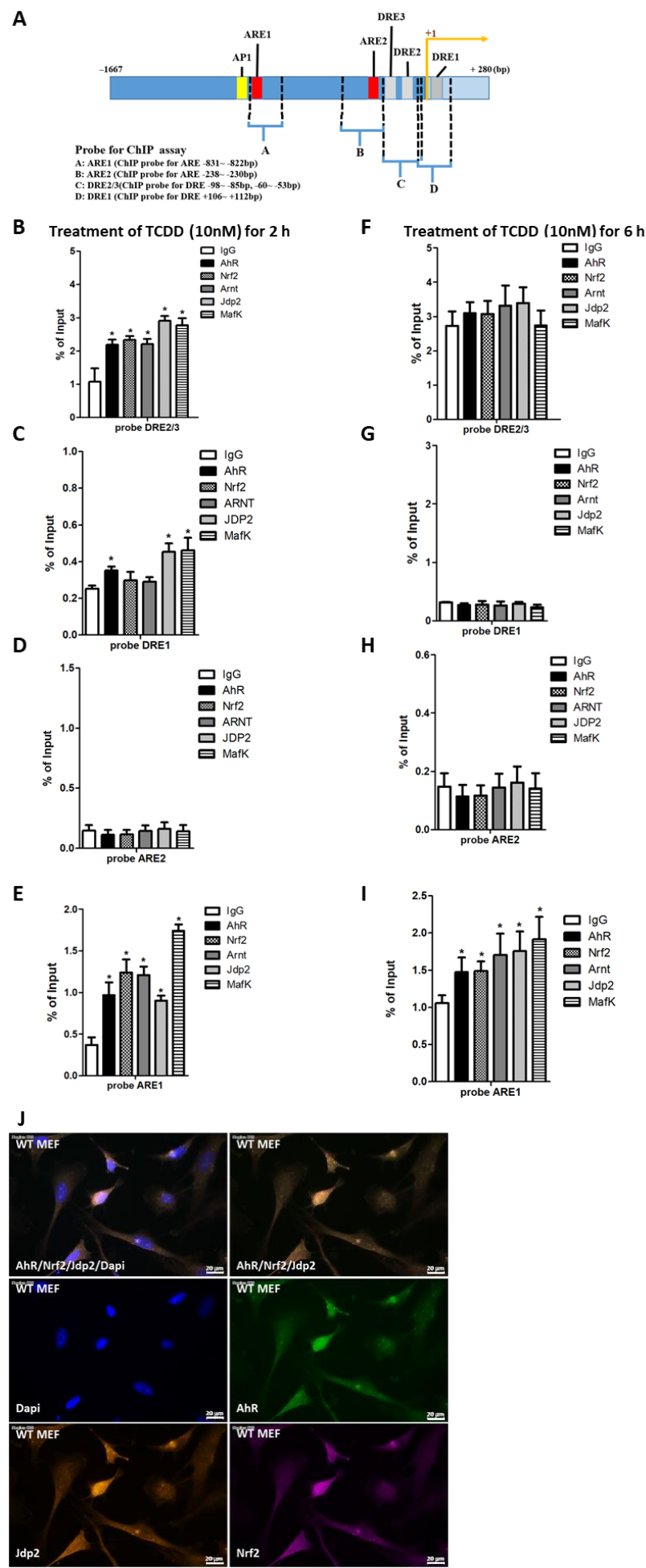

**Figure 4.**

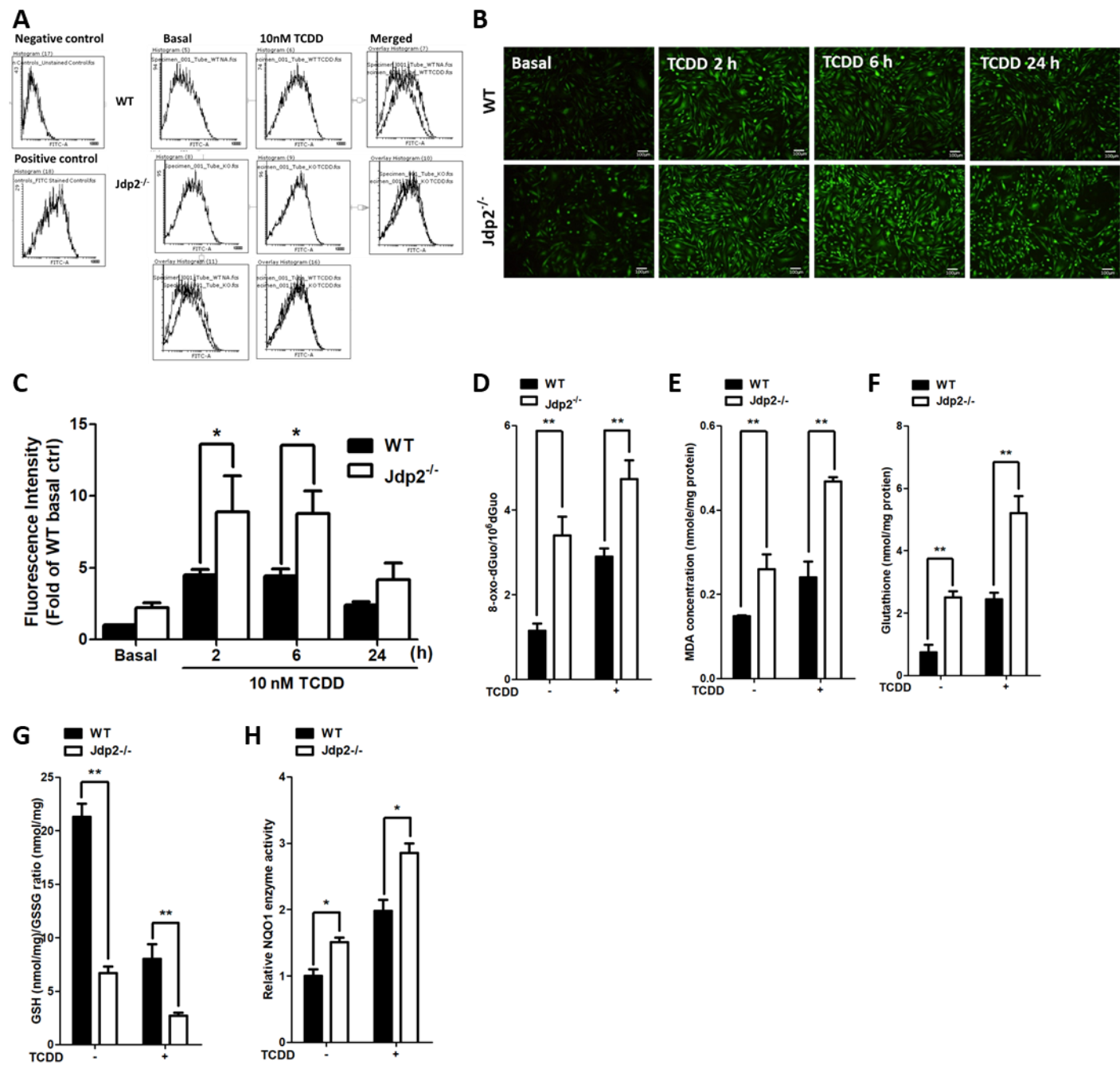

**Figure 5.**

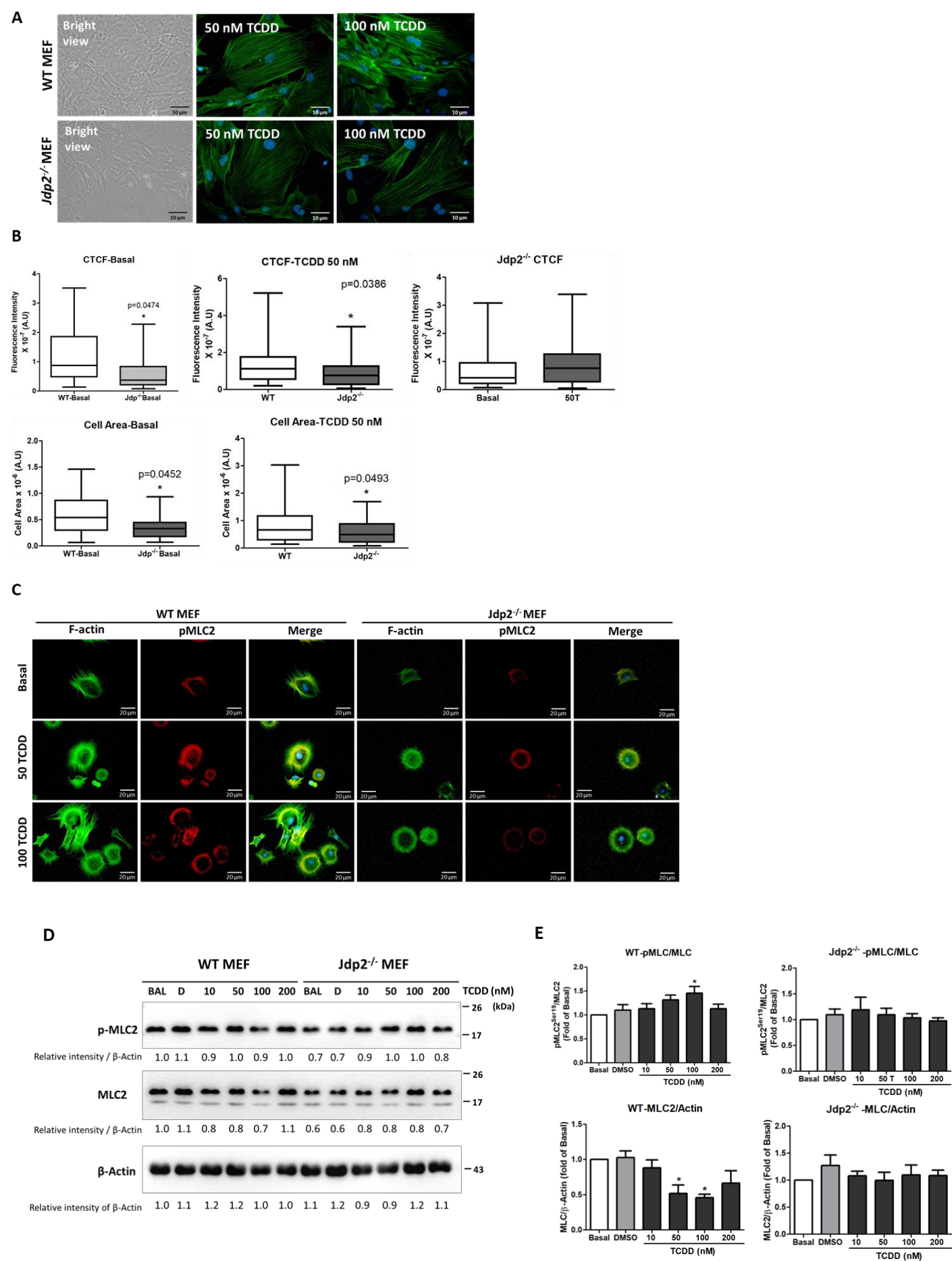

Figure 6.

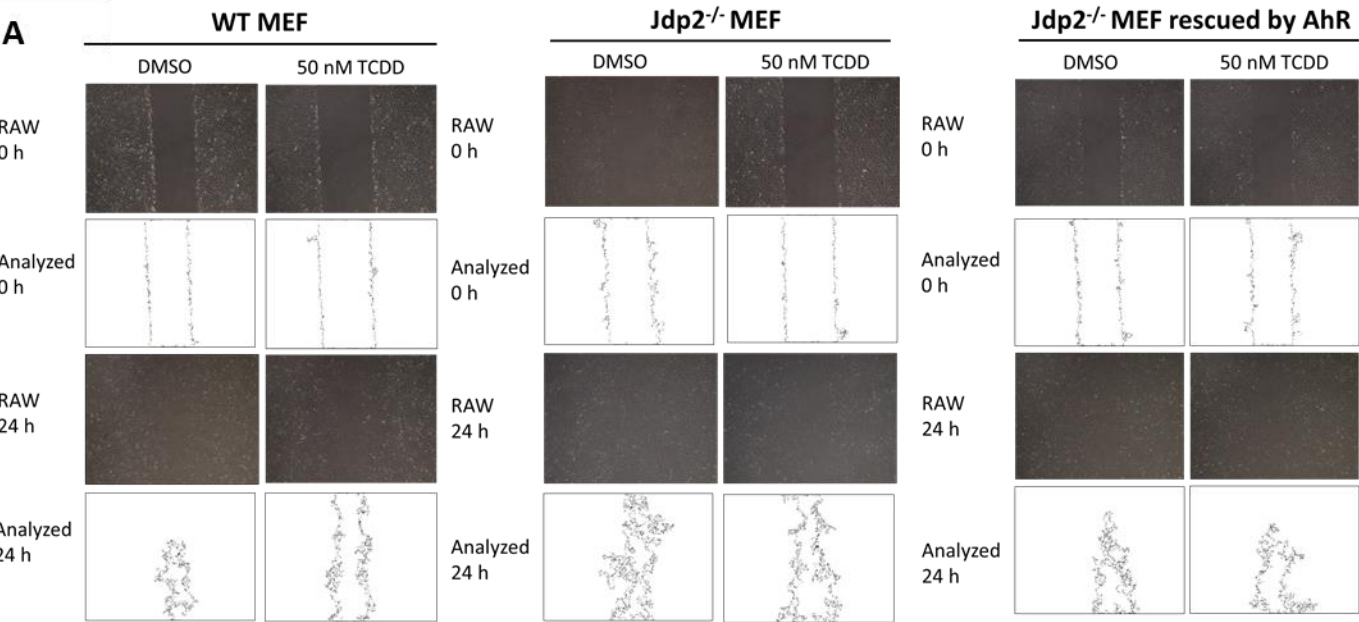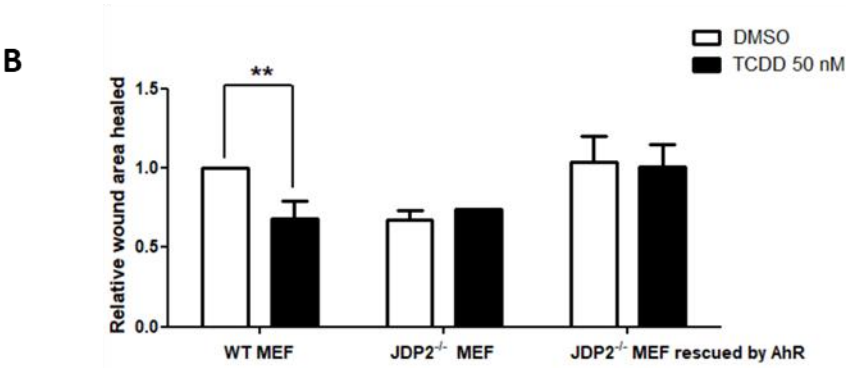

**Figure 7.**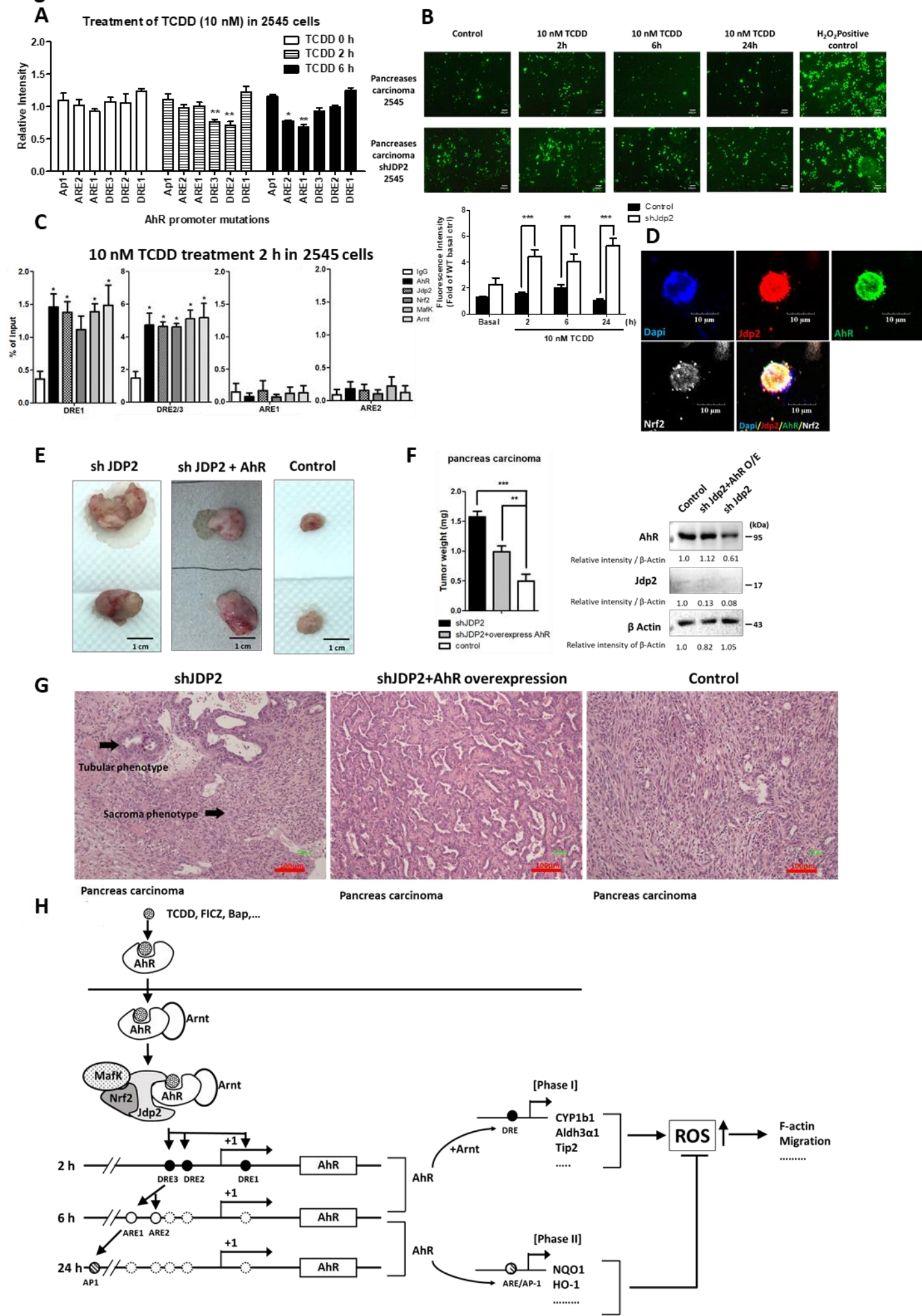

Supplement: Supplementary file 1 — Additional file 1: Supplementary Figure S1. Luciferase activity driven by the Cyp1b1 promoter in response to TCDD. Supplementary Figure S2. Characteristics of AhR protein expression and effects of Jdp2 on AhR expression. Supplementary Figure S3. Characterization of AhR promoter activity. Supplementary Figure S4. Interaction of the AhR–Jdp2–Nrf2 axis in nuclei and cytoplasm in WT and Jdp2–/–MEFs in response to TCDD or DMSO exposure for the times indicated. Supplementary Figure S5. Characterization of the DRE2 mutation in the AhR promoter region and expression of the Jdp2 target genes. Supplementary Figure S6. EMSA assay of GST–AhR–basic helix-loop-helix (aHLH) binding to DRE2, DRE3 and ARE1 in vitro. Supplementary Figure S7. Relative mRNA expression of the AhR target genes Aldh3a1 (A), Cyp1b1 (B), and Tiparp (C) after exposure to DMSO and TCDD in WT and Jdp2−/− MEFs. Supplementary Figure S8. Uncropped raw data of western blots which were used in this article. Supplementary Table S1. Antibodies used in this study. Supplementary Table S2. Oligonucleotides. Supplementary Table S3. Mutation of ARE1, ARE2, DRE1, DRE2, and DRE3 in AhR promoter. Supplementary Table S4. siRNAs used in this study. Supplementary Table S5. Experimental models: organisms/strains. Supplementary Table S6. Experimental models: cell line. Supplementary Table S7. Critical commercial assay. Supplementary Table S8. Chemicals, peptides, and recombinant proteins. Supplementary Table S9. Recombinant DNA Supplementary Table S10. Software and algorithms. [file 41232_2023_290_MOESM1_ESM.zip › Inflammation & Regeneration figures 20230601-Final version.pdf]
